# First chance to see: New jumping-spider species from Batanta, Indonesia (Araneae, Salticidae, Euophryini)

**DOI:** 10.3897/zookeys.1293.190325

**Published:** 2026-10-01

**Authors:** Hunor Takács-Vágó, Ádám Sándor Mayer, Krisztián Szabó, Tibor Kovács, Tamás Szűts

**Affiliations:** 1 Diversity and Evolution Research group, Department of Zoology, University of Veterinary Medicine, Budapest, Hungary Diversity and Evolution Research group, Department of Zoology, University of Veterinary Medicine Budapest Hungary; 2 Mátra Museum of the Hungarian Natural History Museum of the Hungarian National Museum Public Collection Centre, Gyöngyös, Hungary Mátra Museum of the Hungarian Natural History Museum of the Hungarian National Museum Public Collection Centre Gyöngyös Hungary

**Keywords:** *
Canama
*, integrative taxonomy, molecular phylogeny, new combination, new species, species discovery

## Abstract

The first spider records of Batanta, Birie, and Arefi islands, Raja Ampat, Indonesia are presented in this study. Three new species in the genus *Canama* Simon, 1903, *Canama horobi***sp. nov**. (male), *Canama limagigi***sp. nov**. (male) and *Canama wanita***sp. nov**. (female) are described. Two Indonesian salticids, originally placed in *Bathippus* Thorell, 1892 and known from their original descriptions only, *Bathippus dentiferellus* Strand, 1911 and *Bathippus seltuttensis* Strand, 1911, are redescribed and illustrated. Due to their morphological similarity to the type species, three new combinations have been proposed: *Canama dentiferellus* (Strand, 1911), **comb. nov**., *Canama seltuttensis* (Strand, 1911), **comb. nov**. and *Canama dilanians* (Thorell, 1881), **comb. nov**. To confirm the phylogenetic placement of the new species, we carried out a small phylogenetic analysis with three genetic markers: 28S, ND1/16S, and COI, with a final alignment of 3314 nucleotides. Both maximum likelihood and Bayesian results confirmed their firm placement within *Canama*. With 10 new sequences, 49 original images and a distribution map.

## Introduction

The Indonesian archipelago stands as one of the biggest biodiversity hotspots of our planet (Myers et al. 2000). Stretching from west of the Malay peninsula till the midline of New Guinea, it has biogeographical significance paired with a high number of species still to discover (Ondo et al. 2024). On the eastern part of the archipelago, just a few kilometres off the New Guinean west coast lies the Raja Ampat Regency, containing about 1500 islands. Batanta is the fourth largest island within that regency and yet avoided the attention of arachnologists. Despite citizen science observations (i.e. iNaturalist) have been recorded, the only actual published data on the arachnofauna is on Amblypygi (Rahmadi and Kojima 2010), thus not a single jumping spider has been recorded from there (World Spider Catalog 2026; Metzner 2026). The lack of data is due to the lack of sampling and scientific exploration rather than absence of biota, a clear example of Linnean and Wallacean shortfalls (Hortal et al. 2015). Few accounts of the zoological exploration of the island just have been published (Kovács et al. 2015, 2025), giving a nice review what has been achieved in the past two decades or so. Recent collections also resulted in a few jumping spiders as bycatch from the island, so we can report the first glimpses of the salticid fauna of Batanta and surrounding smaller islets Arefi and Birie.

In this paper we use an integrative approach to tackle generic placement challenges, and we first focus on taxa that has been recently worked upon. The genus *Canama* Simon, 1903 is relatively easy to recognize, due to the large large chelicerae of males, although a full revision of the genus and its close relative *Bathippus* Thorell, 1892 is still not published. We describe here three new *Canama* species from Batanta and surrounding islands: *C. horobi* sp. nov., *C. limagigi* sp. nov., *C. wanita* sp. nov. and confirm their placement in the genus via morphological characters and a three genes molecular phylogenetic analysis. We redescribe two Indonesian species, *Bathippus dentiferellus* Strand, 1911 and *Bathippus seltuttensis* Strand, 1911. We are proposing three new combinations in *Canama dentiferellus* (Strand, 1911), comb. nov., *Canama seltuttensis* (Strand, 1911), comb. nov., and *Canama dilanians* (Thorell, 1881), comb. nov. based on similarities to the type species in the embolus, the RTA shape and female epigyne.

## Material and methods

### Morphology methods

Preserved specimens were collected and kept in 96% ethanol. Images were obtained with a Tucsen MiChrome camera attached to a Nikon SMZ800 and a Tucsen TrueChrome Metrics camera attached to a Nikon Eclipse E200 compound microscope. Stack images were combined to a multifocal image with Helicon Focus 8 (licensed to T. Szűts). Legs, chelicerae, and male palps were dissected from the specimens with forceps and were photographed and measured separately. Protocol for copulatory organ photography follows Szűts et al. (2020).

Terminology follows Zhang and Maddison (2012). For leg spination, terminology follows Ono (1988). All measurements are given in millimetres. Total length measurements are referred from the anterior margin of the prosoma to the posterior margin of the opisthosoma. Carapace length measurements were taken from the anterior margin to the posterior margin of the prosoma. Measurements were obtained in the ImageJ v. 1.53t (Schneider et al. 2012). The distribution map has been downloaded from OpenStreetMap and images downloaded from Wikipedia (CC BY-SA 3.0).

### Molecular methods

Tissue samples were retrieved from freshly collected specimens and preserved in 96% ethanol until laboratory procedures. Whole genomic DNA was extracted from one or two clipped legs, using standard extraction kits. Three gene fragments were amplified by PCR, following their protocols: (1) the barcoding segment of the protein coding mitochondrial cytochrome c oxidase subunit I (COI) using the LCO1940/HCO2198 primers (Folmer et al. 1994), (2) a partial sequence of the mitochondrial ND1 and 16s RNA genes using the primer pairs LRN12945/N1J12261 (Hedin 1997), (3) the divergent D2 and D3 domains of the nuclear 28s RNA gene (28S) using the primer pairs 28SO/28SC (Hedin and Maddison 2001).

Zhang and Maddison (2013) had used a different primer for COI (C1-J-1718 (forward) Simon et al. 1994 and C1-N-2776 (reverse) Hedin and Maddison 2001); thus, our dataset has several fragments with only partial overlap (~300 bp) for that locus, resulting in missing data across the alignment. To compensate the data heterogeneity which may affect phylogenetic inference, we have included sequences of two extra specimens of *Bathippus madang* Zhang & Maddison, 2012 (SAJ304 – three loci, SAJ308 – 16S) as control besides the voucher JXZ143.

PCR was carried out at the Department of Zoology of the University of Veterinary Medicine Budapest, and capillary electrophoresis was done using an ABI 35000xL sequencer (Applied Biosystems, Foster City, CA, USA) at commercial Biomi Services (Gödöllő, Hungary).

Newly generated sequences were edited using Gap4 of the Staden Package (Staden et al. 2000) and deposited in GenBank. Legacy data sequences (Table 1) were downloaded and combined our own sequences and managed in Mesquite v. 4.02 (Maddison and Maddison 2025). Each locus was aligned separately in the Mesquite built-in MAFFT multiple alignment tool ‘L-INS-I’ option with the ‘--localpair --maxiterate 1000' command.

**Table 1.** List of the GenBank accession numbers of taxa used in this study.

**Taxon**	**Voucher code**	**16S**	**28S**	**COI**
*Bathippus cf. directus*	JXZ259	KC615824	KC615396	KC615651
*Bathippus directus*	JXZ205	KC615823	KC615395	KC615650
*Bathippus gahavisuka*	JXZ228	KC615825	KC615397	KC615652
*Bathippus korei*	JXZ284	KC615826	KC615398	KC615653
*Bathippus macrognathus*	JXZ372	KC615835	KC615407	—
***Bathippus madang***	**SAJ308**	**Pending**	—	—
***Bathippus madang***	**SAJ304**	**Pending**	**PZ938007**	**PZ934522**
*Bathippus madang*	JXZ143	KC615840	KC615413	KC615662
*Canama cf. forceps*	JXZ210	KC615841	KC615414	KC615663
*Canama extranea*	JXZ258	KC615828	KC615400	KC615654
*Canama fimoi*	JXZ265	KC615827	KC615399	—
*Canama hinnulea*	JXZ089 + 161	KC615842	KC615415	—
***Canama horobi***	SAJ455	**Pending**	**PZ938009**	—
*Canama triramosa*	JXZ277	KC615922	KC615497	—
***Canama wanita***	SAJ148	—	—	**PZ934521**
***Canama limagigi***	SAJ309	**Pending**	**PZ938008**	**PZ934523**
*Coccorchestes cf. aiyura*	JXZ289	KC615879	KC615452	KC615683
*Coccorchestes cf. inermis*	JXZ139	KC615881	KC615454	KC615684
*Viribestus suyanensis*	JXZ261	KC615912	KC615487	KC615700

The final alignment of the three-loci dataset (28S with 1169 nts, ND1/16S with 951 nts and COI with 1194 nts, altogether 3314 positions; see Suppl. material 1) was further analysed on the NGPhylogeny server (Lemoine et al. 2019).

Each locus was treated as a separate partition; the substitution models were selected via SMS (Lefort et al. 2017). Phylogenetic trees were calculated in two separate analyses, using IQ-TREE v. 3.0.1 (Trifinopoulos et al. 2016), performing 1000 ultrafast bootstrap replicates (Guindon et al. 2010) (Fig. 12) and MrBayes (Huelsenbeck and Ronquist 2001) (Suppl. material 1: fig. S1), both running on the NGPhylogeny server (Lemoine et al. 2019). Both analyses were running with default settings, using the GTR+G+I substitution model for each locus. Both phylograms were edited with iTOL (Letunic and Bork 2024). All sequences, final alignment and raw trees are available in the Suppl. material 1.

Abbreviations used:

**PLE** Posterior lateral eyes;

**RTA** Retrolateral tibial apophysis;

**Fm** Femur;

**Pt** Patella;

**Tb** Tibia;

**Mt** Metatarsus;

**d** dorsal;

**v** ventral;

**pr** prolateral;

**rt** retrolateral;

**ap** apical;

**HNHM** Hungarian Natural History Museum of the Hungarian National Museum Public Collection Centre, Budapest, Hungary (Curator: Edit Horváth).

## Results

### Taxonomy

**Family Salticidae Blackwall, 1841**

**Tribe Euophryini Simon, 1901**

#### Genus Canama

Taxon classificationAnimaliaAraneaeSalticidae

Simon, 1903

C7416591-5C51-5F53-8778-249430A57146

Figs 1, 2, 3, 4, 5, 6, 7, 8, 9, 11C, D

##### Type species.

*Salticus forceps* Doleschall, 1859 by original designation.

##### Diagnosis.

**Figure 1.**
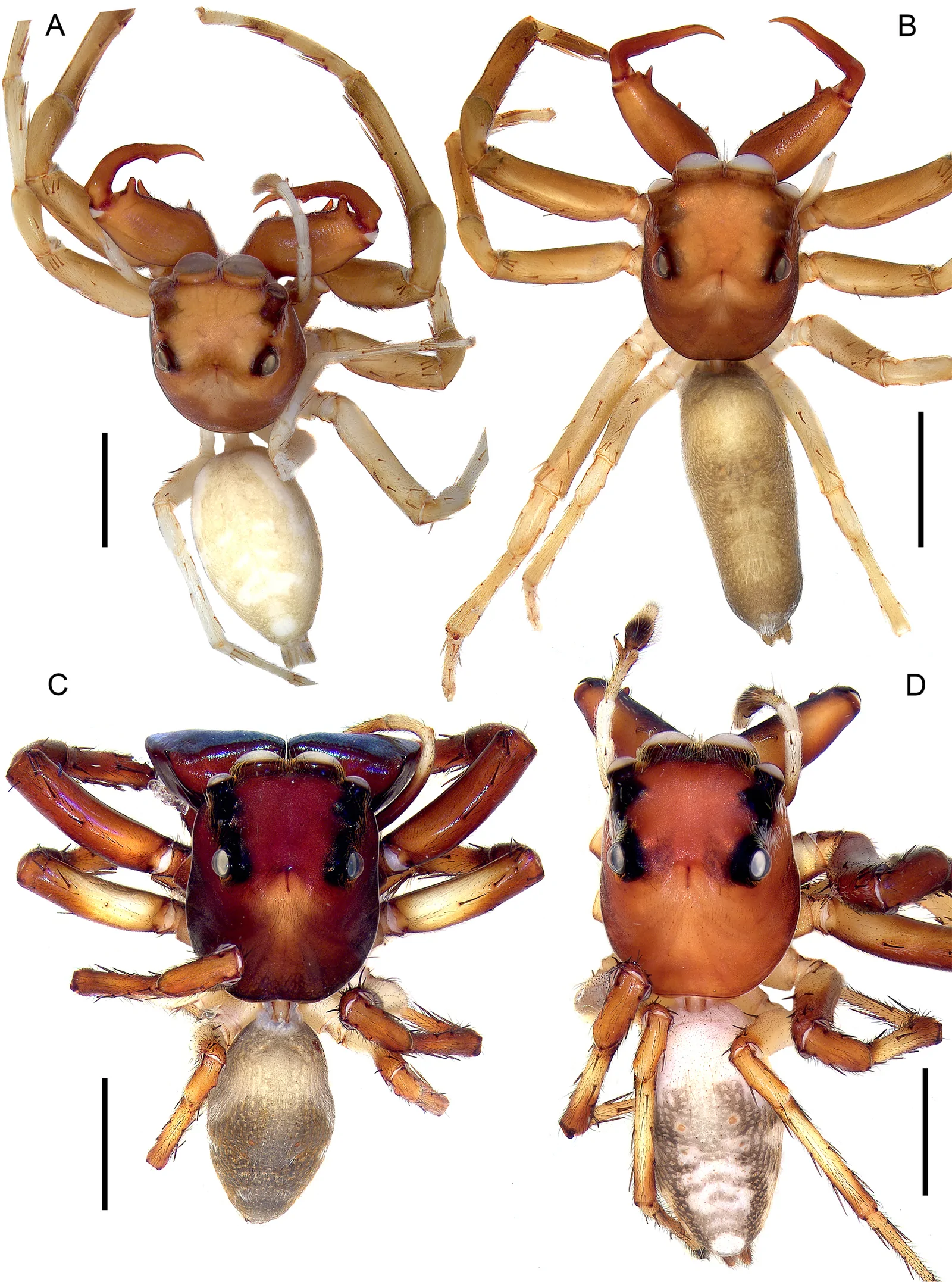
Habitus of *Canama* males, dorsal views. **A**. *C. dentiferellus* holotype; **B**. *C. seltuttensis* holotype; **C**. *C. horobi* holotype; **D**. *C. limagigi* paratype. Scale bars: 2.0 mm.

The genus can be identified by and differentiated from the closely related *Bathippus* based on the combination of the following characters: males with exaggerated chelicerae (Fig. 1) which are usually thicker and wider than longer (vs thin and usually two or three times the length of the prosoma in *Bathippus*), thin embolus that coils usually more than a circle (Fig. 5; vs thicker, shorter, thread-like embolus that coils usually only one circle in *Bathippus*), the plane of the embolus closes a ~30° angle perpendicular to the plane of the cymbium (vs the plane of the embolus is parallel to the plane of the cymbium in *Bathippus*), the embolus coils in a wide circle (vs embolus narrowly coils in *Bathippus*) and the well-developed, long, distally curved RTA (vs small, finger-like and straight RTA in *Bathippus*). Females with less swollen, round spermathecae with median ridge and genital openings situated below the spermathecae, sometimes with a small epigynal window (Fig. 9; vs female epigyne of *Bathippus* with large window, swollen spermathecae, and the opening to the copulatory duct is at the anterior or median of the vulva; for more details see Zhang and Maddison 2012).

**Figure 2.**
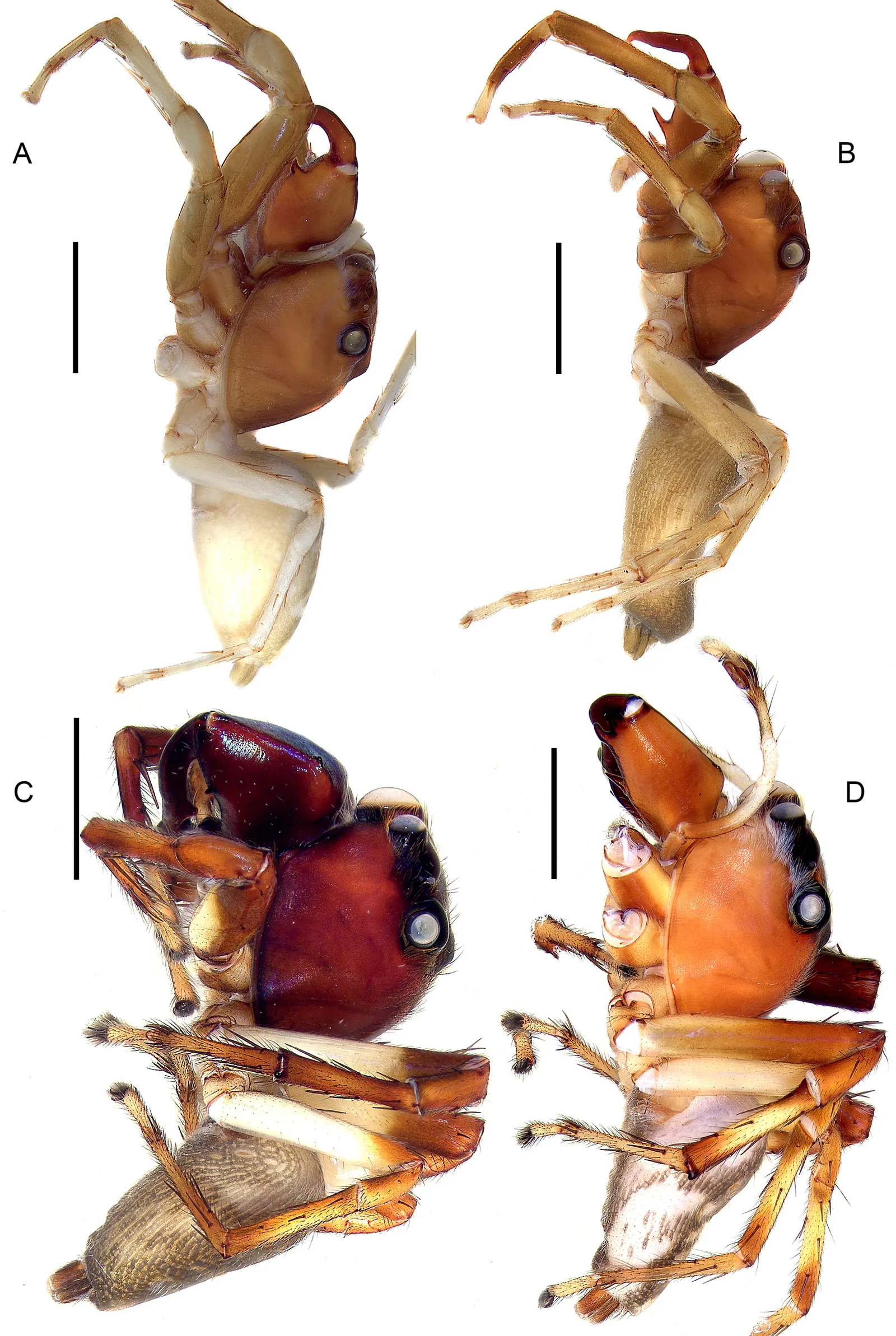
Habitus of *Canama* males, lateral views. **A**. *C. dentiferellus* holotype; **B**. *C. seltuttensis* holotype; **C**. *C. horobi* holotype; **D**. *C. limagigi* paratype. Scale bars: 2.0 mm.

**Figure 3.**
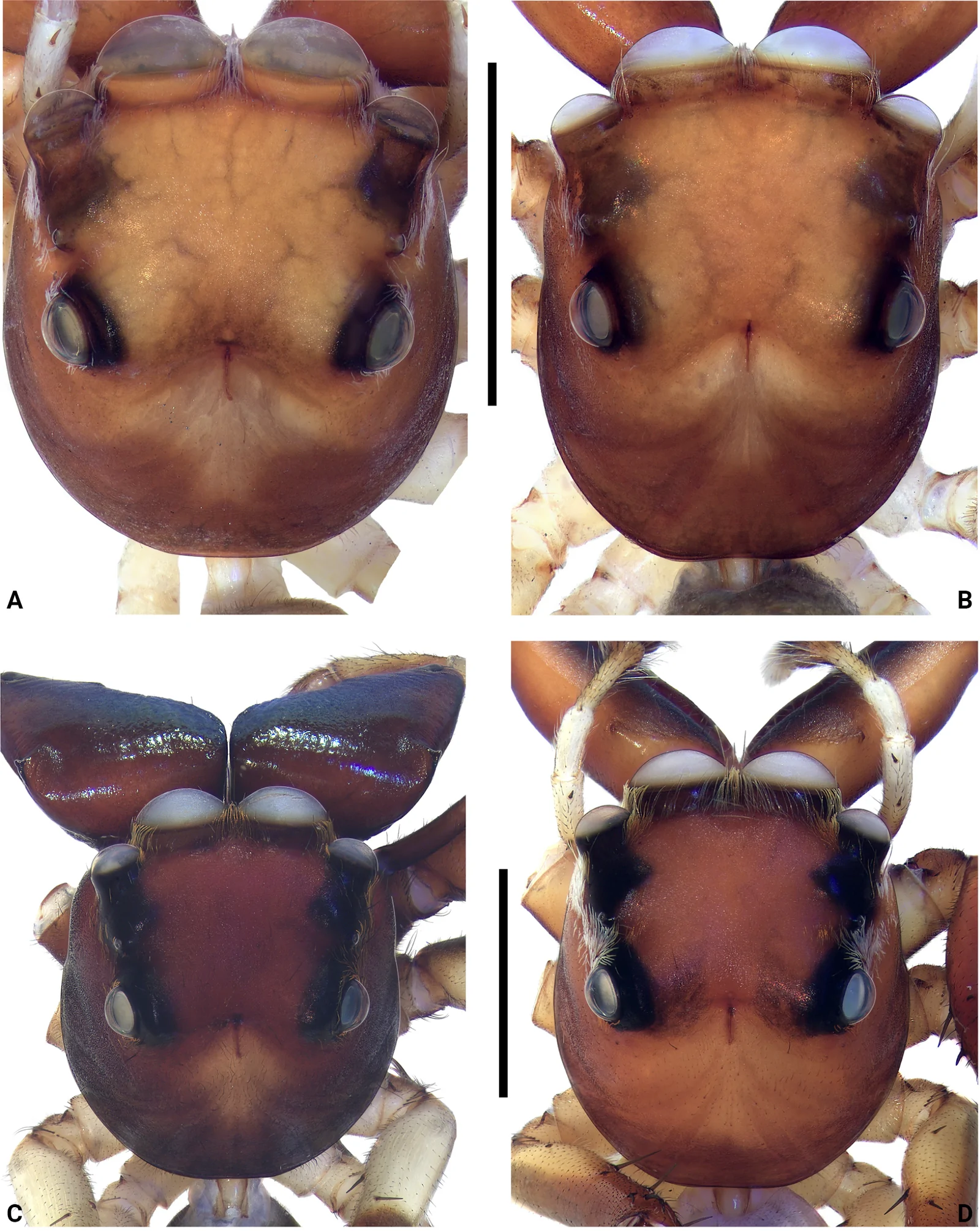
Carapaces of *Canama* males, dorsal views. **A**. *C. dentiferellus* holotype; **B**. *C. seltuttensis* holotype; **C**. *C. horobi* holotype; **D**. *C. limagigi* paratype. Scale bars: 2.0 mm.

##### Description.

**Figure 4.**
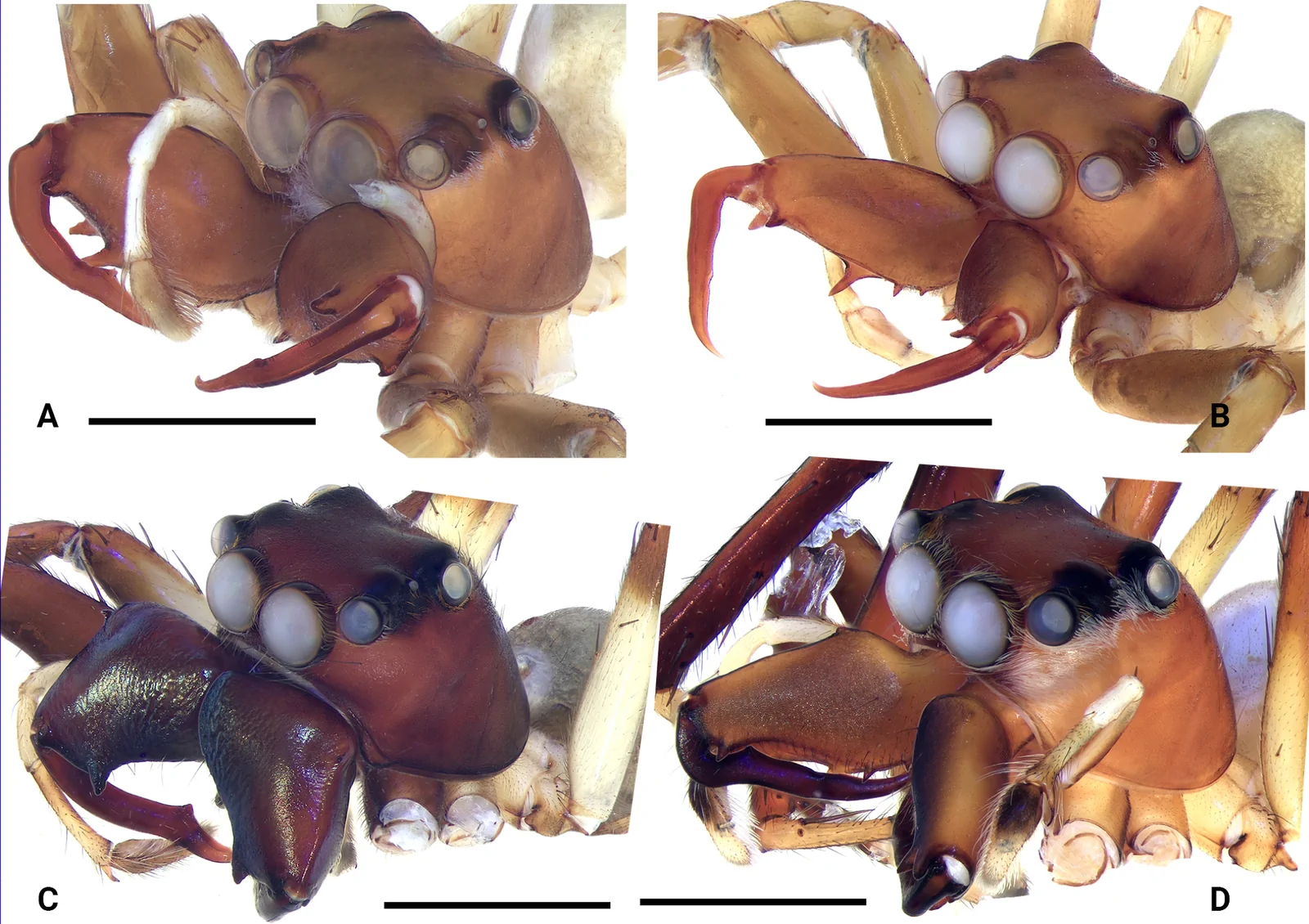
Prosoma of the *Canama* males, fronto-lateral view. **A**. *C. dentiferellus* holotype; **B**. *C. seltuttensis* holotype; **C**. *C. horobi* holotype; **D**. *C. limagigi* paratype. Scale bars: 2.0 mm.

**Figure 5.**
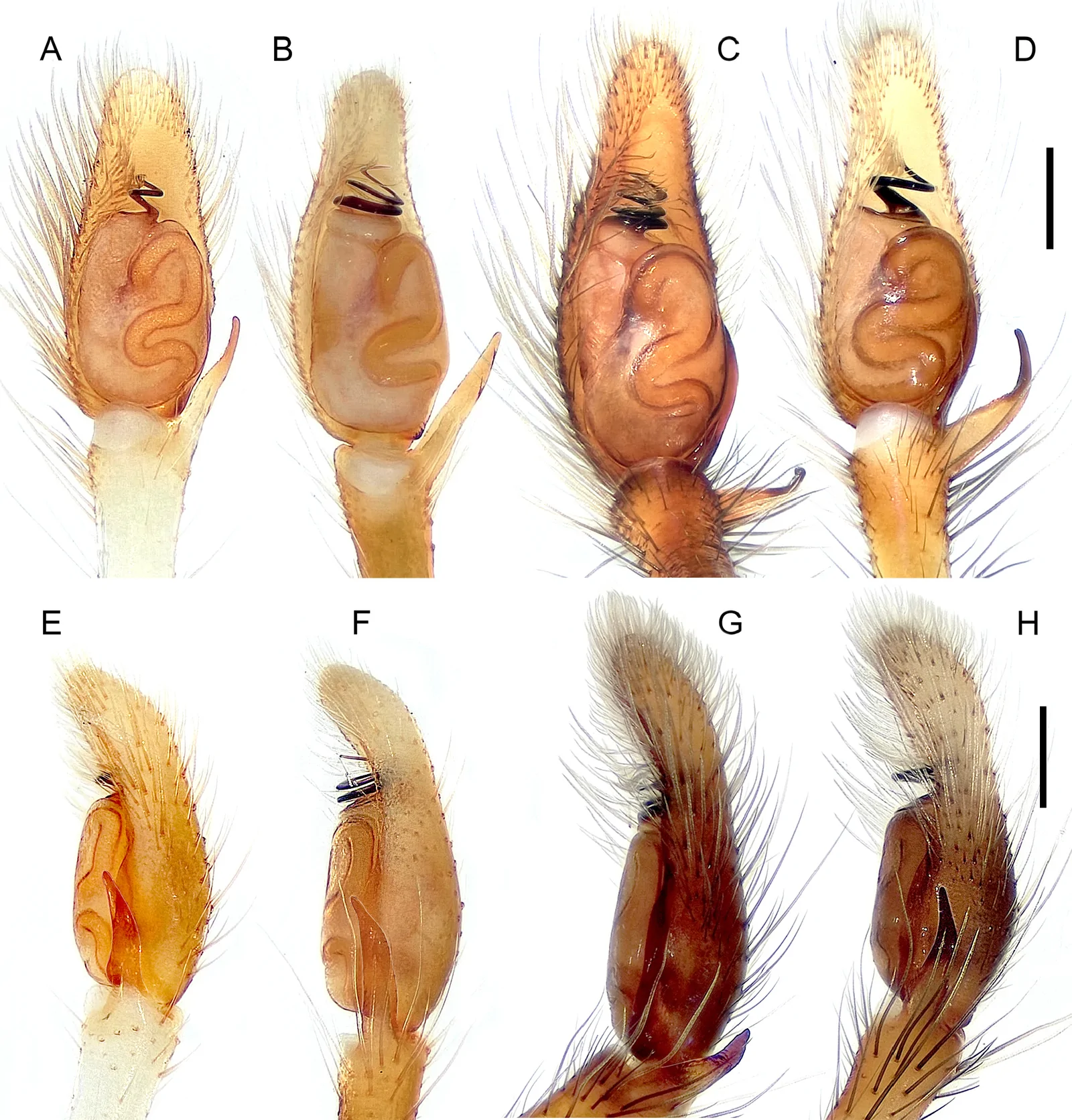
Male palps of the examined *Canama* males. **A, E**. *C. dentiferellus* holotype; **B, F**. *C. seltuttensis* holotype; **C, G**. *C. horobi* holotype; **D, H**. *C. limagigi* paratype. **A–D** Ventral view; **E–H** Retrolateral view. Scale bars: 0.25 mm.

Medium-sized to large spiders; males usually with large, elongated or wide chelicerae, similar to *Bathippus* species (Figs 1, 4) and with teeth varying in shape and size. Females with unmodified chelicerae, usually with a single bicuspid retromarginal tooth or two retromarginal teeth (Fig. 8E). Male palpal bulb elongated, oval, and without proximal tegular lobe (Fig. 5). Embolus thin, long, twisted perpendicular to axis of cymbium. Spermophore wide, usually occupying about ^2^/_3_ width of bulb. Retrolateral tibial apophysis (RTA) large, long, simple, usually with a curve at distal end. Spermathecae rather coiled, continuous with copulatory duct, usually not swollen (Zhang and Maddison 2012). Genital openings situated below spermathecae, relatively far from each other.

##### Composition.

So far, nine species have been described from Australia, the Pacific islands, Malaysia, and Papua New Guinea, to which we add six species (three species new to science, *C. horobi* sp. nov., *C. limagigi* sp. nov., and *C. wanita* sp. nov., and three new combinations, *C. dentiferellus* comb. nov., *C. dilanians* comb. nov., and *C. seltuttensis* comb. nov.).

##### Remarks.

There was a debate about the clear delimitation of *Bathippus* and *Canama* at the late 1980s. Prószyński (1987) suggested that *Canama* is a junior synonym of *Bathippus*, based on the similarities. Davies and Żabka (1989) disagreed with Prószyński, despite that they based their arguments on species now belonging to *Parabathippus* Zhang & Maddison, 2012. They suggested the separation of *Bathippus* and *Canama* based on the differences in the structure of the chelicerae and epigyne. Zhang and Maddison (2013, 2015) showed that both *Canama* and *Bathippus* form monophyletic groups and thus argued to keep these genera separate; these authors described the genus *Parabathippus* to accommodate about 10 species from South-East Asia and Indonesia previously placed in *Bathippus*.

**Figure 6.**
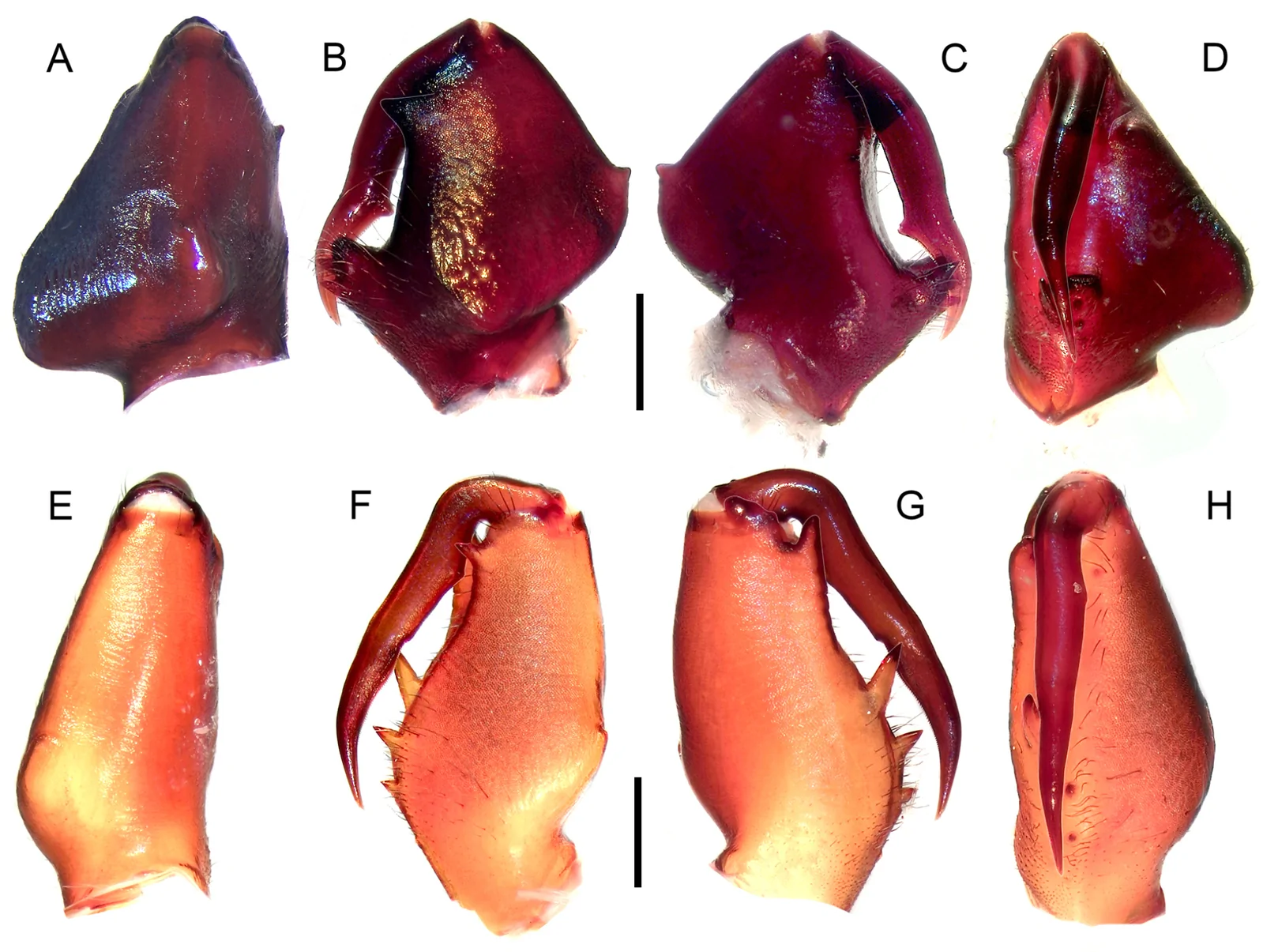
Chelicerae of male *Canama horobi* and *Canama limagigi*. **A–D**. *C. horobi*; **E–H**. *C. limagigi*. **A, E**. Ectal view; **B, F**. Prolateral view; **C, G**. Retrolateral view; **D, H**. Mesal view. Scale bars: 0.5 mm.

**Figure 7.**
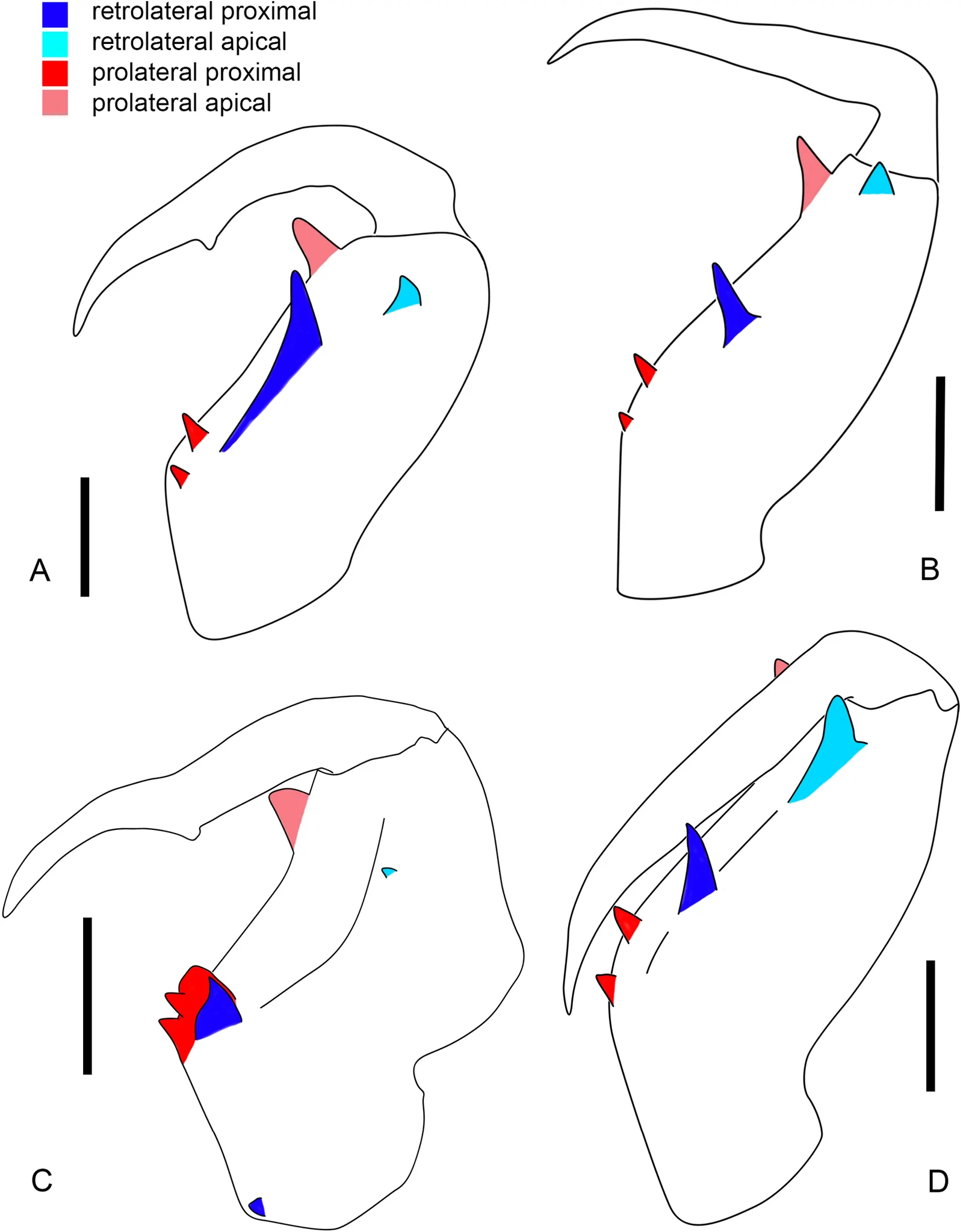
Cheliceral dentitions of *Canama* males, ventral-mesal view. **A**. *C. dentiferellus*; **B**. *C. seltuttensis*; **C**. *C. horobi*; **D**. *C. limagigi*. Scale bars: 0.5 mm.

Our strategy to place the new species was integrative: we used similarity of morphology to the type species—*Salticus forceps* Doleschall, 1859—such as the elongated but bulky chelicerae of the male and the shape of the male embolus and RTA. We used three molecular markers to test whether the morphological placement of *C. horobi* sp. nov., *C. limagigi* sp. nov., and *C. wanita* sp. nov. is reinforced by molecular data. Both morphological and molecular data suggest that these species belong in *Canama* (Figs 5, 7, 11). We redescribe two species—*Bathippus dentiferellus* Strand, 1911 and *Bathippus seltuttensis* Strand, 1911—and both also share with the new *Canama* species the same shape of RTA and embolus (Fig. 5), together with the bulky chelicerae (Figs 6, 7); thus, these two species are transferred to *Canama*. *Bathippus dilanians* (Thorell, 1881) is well illustrated in the literature (Prószyński, 1984) also shows great similarity to these species (in fact the new *Canama* species are often diagnosed against it) and, thus, is also transferred to *Canama*.

**Figure 8.**
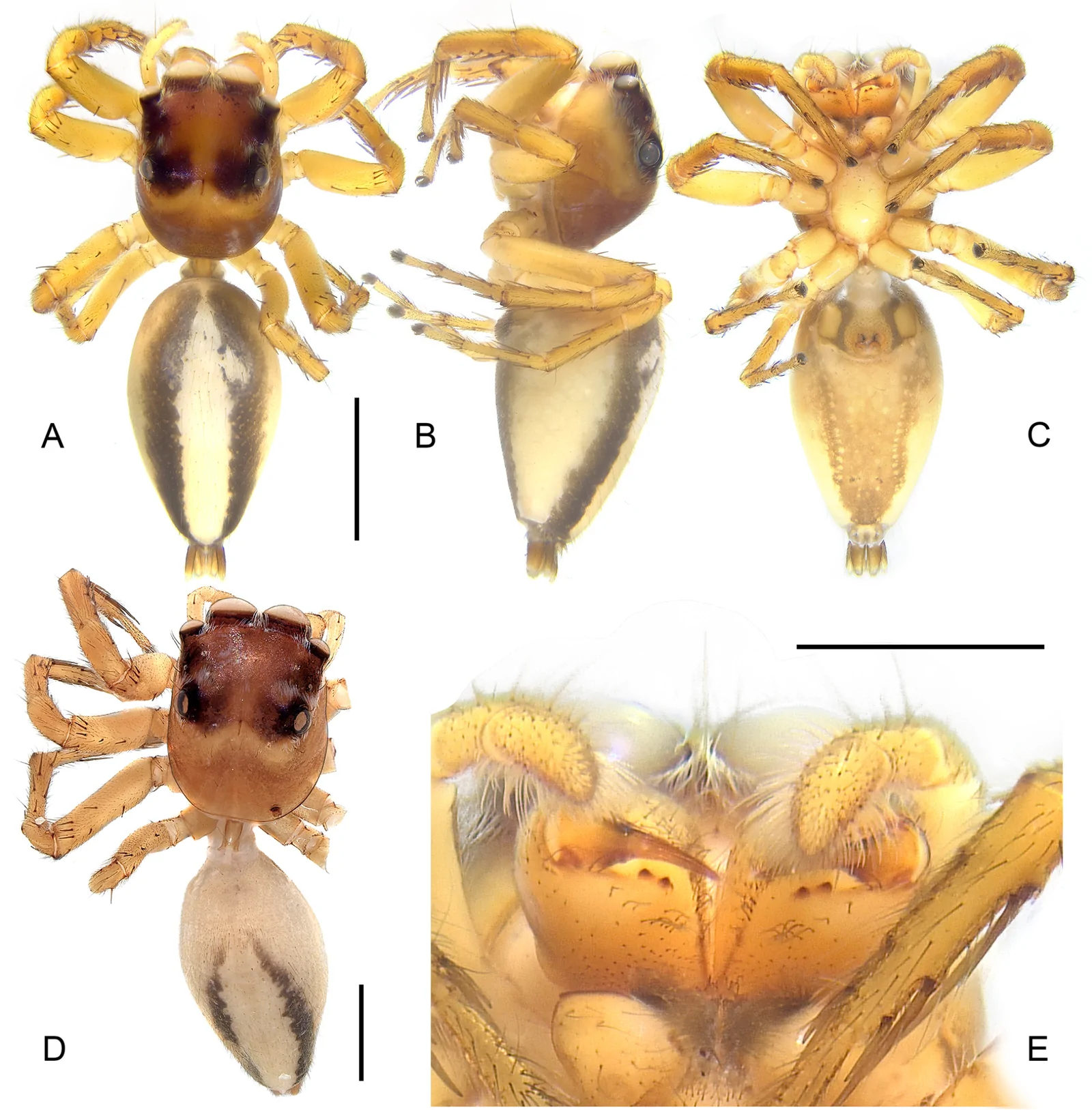
Habitus of *Canama wanita* female. **A–C**. Female paratype (HNHM11656) from Batanta Island. **A**. Dorsal view; **B**. Lateral view; **C**. Ventral view. **D, E**. Female holotype (HNHM9284) from Arefi Island. **D**. Habitus dorsal view; **E**. Cheliceral dentition, ventral view. Scale bars: 2.0 mm.

#### Canama dentiferellus

Taxon classificationAnimaliaAraneaeSalticidae

(Strand, 1911) comb. nov.

61ECDCB5-830B-5AA1-B2D9-AB0F2B903610

Figs 1A, 2A, 3A, 4A, 5A, E 7A

Bathippus dentiferellus Strand, 1911: 187, pl. 4, fig. 15, pl. 6, fig. 81.—Prószyński 1984: 8.

##### Material examined.

***Holotype* male** from Indonesia • Seltutti, Kobroor, Aru Island; 29 April 1908; coll. H. Merton; Senckenberg Naturmuseum Frankfurt 2421.

##### Diagnosis.

The palp of *C. dentiferellus* has two, tight loops of the embolus and is similar to those of *C. seltuttensis*, *C. forceps*, and *C. dilanians*. It can be easily differentiated from *C. dilanians* by the shape of the RTA, being less curved (having a strong curve in the distal end in the case of *C. dilanians*) and being even in width (base of RTA is wider in *C. dilanians*). The RTA of *C. dentiferellus* has less significant apical narrowing and there is no apical hook (Fig. 5A, E), whereas *C. dilanians* has an RTA with a wide basal part and a distal hook. The chelicera of *C. dentiferellus* has only a small cusp near the distal end of the fang; *C. dilanians* (Prószyński 1984: 8) has a large tooth.

*Canama dentiferellus* (Fig. 5A, E) also shows similarities to *C. seltuttensis* (Fig. 5B, F) but can be differentiated by the tighter embolic coiling (Fig. 5A vs wider coils in *C. seltuttensis* about two times wider; Fig. 5B). The chelicerae also differ; *C. dentiferellus* has a wide retrolateral basal tooth (Fig. 7A) versus a thin retrolateral tooth in *C. seltuttensis* (Fig. 7B).

##### Redescription of male holotype.

Habitus shown in Figs 1A, 2A, 3A. Total length 7.10; carapace length 3.41; width 2.77; abdomen length 3.69; width 2.20; chelicera length 2.21; width 1.02. Prosoma round in dorsal view, dark coloured, with lighter markings and blackish net-like markings between eyes and thoracic hump (Figs 1A, 2A, 3A, 4A). PLE small (Figs 1A, 2A). Chelicerae (Figs 4A) light brown, relatively short (Fig. 1A), wide (Figs 1A, 2A, 4A). Paturon with three large posterior teeth: one each on promargin and retromargin sides, and one in middle (Fig. 7A); middle tooth longer, angled, closing a 20° angle with paturon (Fig. 7A); these three teeth form a triangle in ventral view. Paturon with two small anterior teeth on promargin (Fig. 7A). Fang with a cusp near distal end (Figs 1A, 2A, 7A), slightly curved on distal end (Figs 5A, 7A). Opisthosoma elongated, narrow (Figs 1A, 2A), and lighter coloured, with darker spots on dorsal part (Fig. 1A). Legs long and slender; first legs more robust than others (Fig. 1A). Measurements of legs: I. 10.00, II. 7.91, III. 9.69, IV. 7.90. Spination of legs: Leg I. Fmd 0-1-1-4ap; Ptpr 1, rt 1; Tbv 2-2-0-2, pr 1-0-1-1, rt 1-0-1-1-0; Mtv 2-0-2, pr 1-0-1ap rt 1-0-1ap. Leg II. Fmd 0-1-1-4ap, pr 1, rt 1; Ptpr 1, rt 1; Tbv 2-1-1-2ap, pr 0-1-0-1-1-0, rt 1-1-1; Mtv 2-0-2, pr 1-0-1, rt 1-0-1. Palp (Fig. 5A, E) with a retrolateral loop of spermophore wide and occupying about ^1^/_2_ width of bulb (Fig. 5A). Embolus coiled for about two complete circles, narrow, tubular, and dark (Fig. 5A). RTA long wide, and flat, narrowing in a curve until the distal end (Fig. 5E).

**Female**. Unknown.

##### Distribution.

Known only from the type locality (Fig. 10A).

#### Canama dilanians

Taxon classificationAnimaliaAraneaeSalticidae

(Thorell, 1881) comb. nov.

4623BA76-D2FC-5FF8-B533-F5B2847F2E1F

Plexippus dilanians Thorell, 1881: 548.
Dudasia fissidentata Strand, 1911: 190, pl. 4, fig. 18, pl. 6, fig. 87.
Bathippus dilanians —Prószyński ,1984: 6–8.

##### Diagnosis.

**Figure 9.**
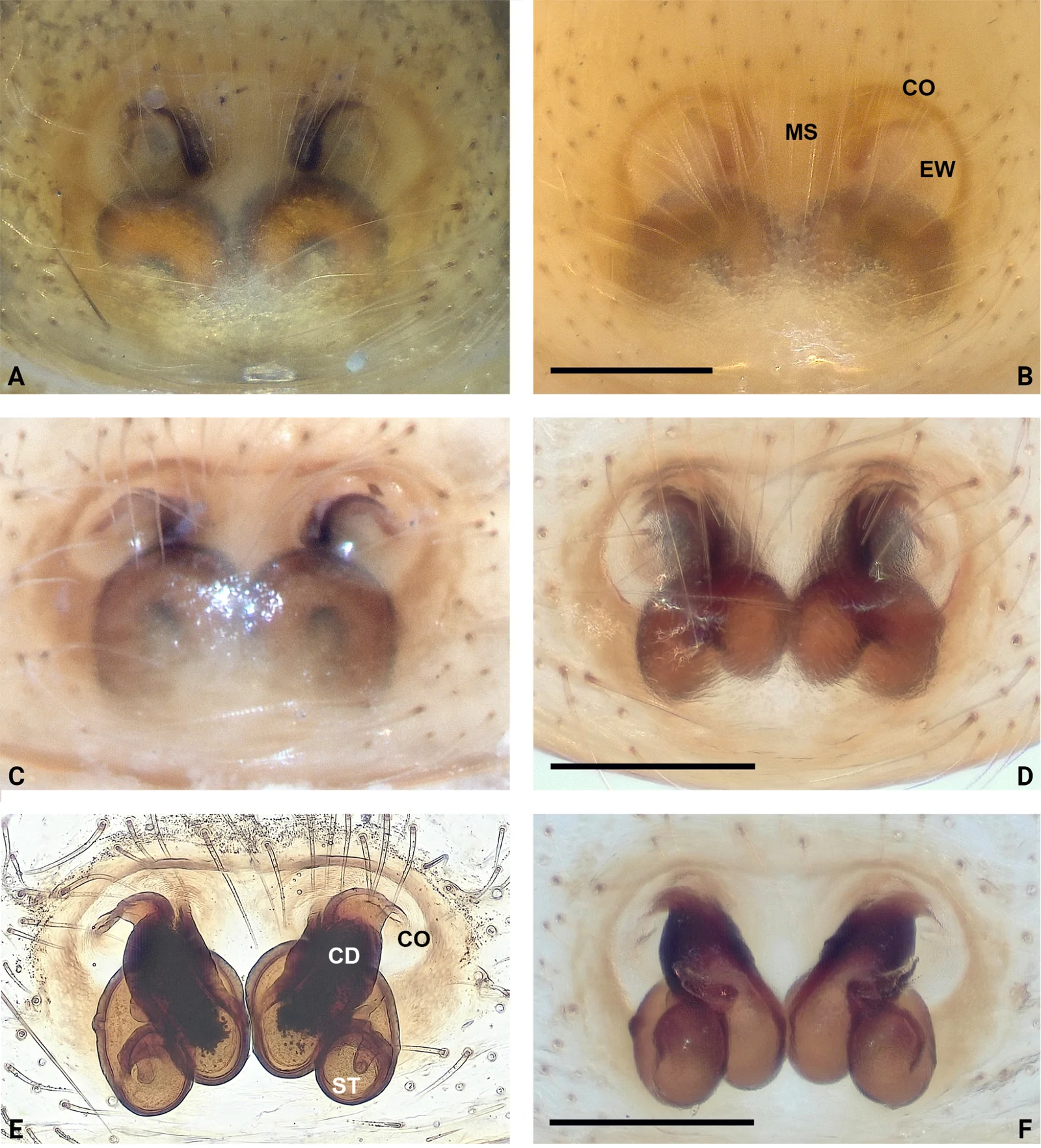
Female copulatory organ of *Canama wanita*. **A**. Paratype female (HNHM11656) from Batanta Island, intact epigyne, ventral view; **B**. Paratype female (HNHM11464) from Birie Island, intact epigyne, ventral view; **C**. Holotype female (HNHM9284) from Arefi Island, intact epigyne, ventral view; **D**. Same, cleaned epigyne, ventral view; **E**. Same, cleaned vulva in methyl-salicylate, dorsal view; **F**. Same, cleaned vulva, dorsal view. Scale bars: 0.25 mm. Abbreviations: CO = copulatory opening, MS = median septum, EW = epigynal window, CD = copulatory duct, ST = spermatheca.

The palp of *C. dilanians* can be easily differentiated from congeners by the RTA of 45° angle and with a strong hook shaped distal curve. This is similar to that of *C. limagigi*; however, *C. dilanians* can be differentiated by the narrow circles of the coiled embolus, occupying about ^1^/_3_ of the bulb width (vs about half of the width of embolus in *C. limagigi*; Fig. 5D). In *C. dilanians* the embolus forms a narrow 1.5 coil. Females can be recognised by the bean-shaped sperm ducts well visible through the cuticle. The sperm ducts are similar to those of *C. wanita* (Fig. 9A–C), but they are only slightly bent in *C. dilanians* (Prószyński 1984: 7) but strongly bent in *C. wanita* (Fig. 9A–C). Male chelicera can be recognised by the ventral extension of the fang (Prószyński 1984: 8).

**Male**. Prószyński 1984: 6–8. Palp (Prószyński, 1984: 7) with an embolus coiled for about 1.5 turn, narrow, tubular, and dark (Fig. 5A). RTA long, wide, and flat, narrowing in a curve until the distal end.

**Female**. Prószyński, 1984: 6–8.

##### Distribution.

**Figure 10.**
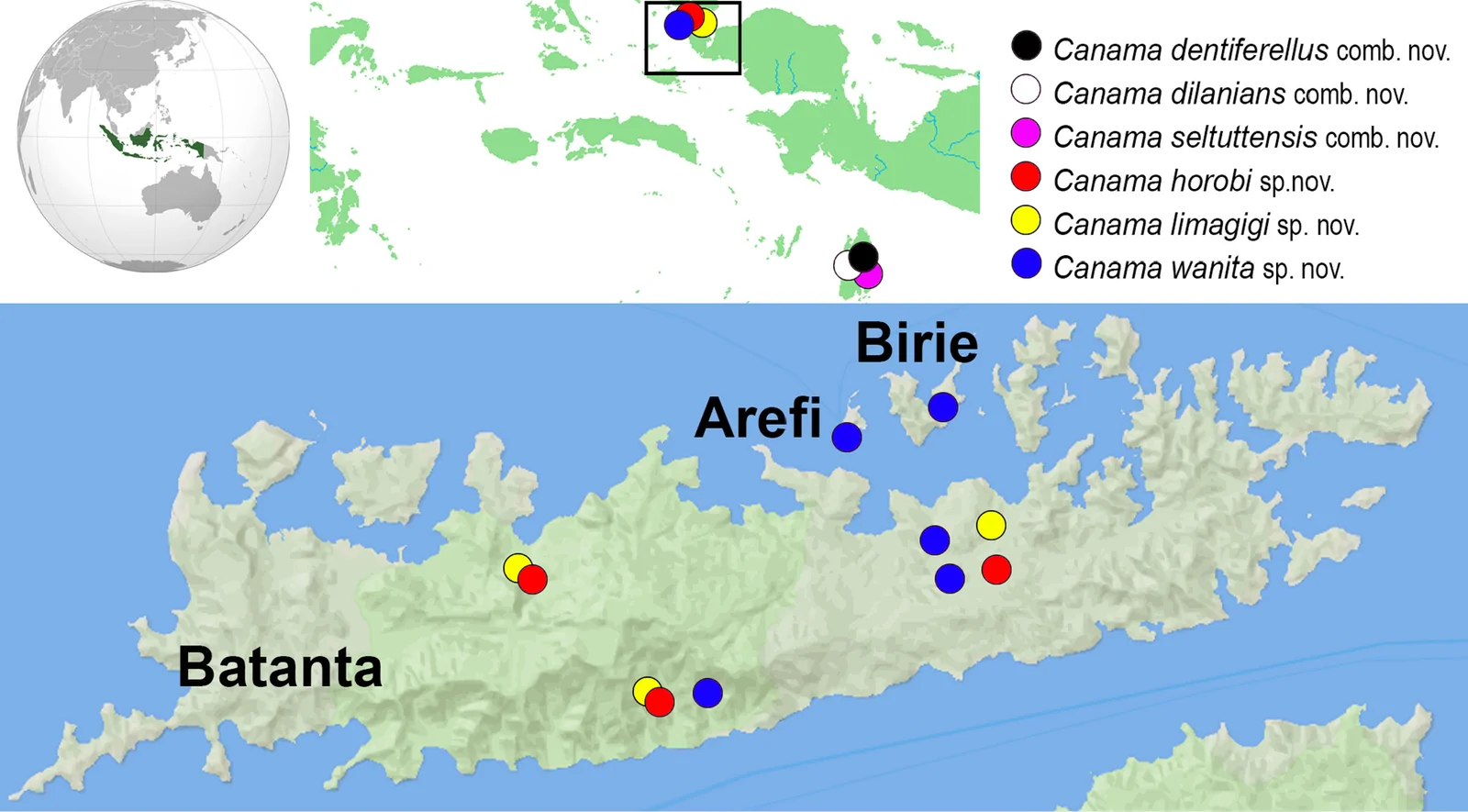
Localities of the treated *Canama* species in Batanta and surrounding islands.

Known only from Aru Island (Fig. 10).

#### Canama seltuttensis

Taxon classificationAnimaliaAraneaeSalticidae

(Strand, 1911) comb. nov.

8C57BB4E-015E-5673-85C5-076205F7800F

Figs 1B, 2B, 3B, 4B, 5B, 5F, 7B.

Bathippus seltuttensis Strand 1911: 188, pl. 6.

##### Material examined.

***Holotype* male** from Indonesia • Seltutti, Kobroor, Aru Island; 3 May 1908; coll. H. Merton; Senckenberg Naturmuseum Frankfurt 2427.

##### Diagnosis.

The palp of *C. seltuttensis* can be recognized by the strong, straight RTA (Fig. 5F), which has a slight bend at the distal end. The chelicera of *C. seltuttensis* has a small hump near the distal end of the fang (Fig. 7B). The paturon of the chelicera bears a straight, pointed, median retromarginal tooth, and one tooth on the distal end of the retromargin.

##### Redescription of male holotype.

Habitus shown in Figs 1B, 2B, 3B. Total length 7.13; carapace length 3.00; width 2.36; abdomen length 4.13; width 1.69; chelicerae length 1.67; width 0.75. Prosoma round in dorsal view and darkly coloured, with lighter markings between eyes and thoracic hump (Figs 1B, 2B, 3B). PLE small (Figs 1B, 3B). Chelicerae (Figs 1B, 2B, 4B, 7B) light brown, short, and wide (Figs 1B, 2B, 4B). Paturon with three posterior teeth about equal in size: two each on retromargin and promargin, and one in middle of paturon on retromargin side (Fig. 7B); these teeth form an elongated triangle in ventral view. Paturon with two small promargin teeth on anterior side. Fang with a small hump near distal end (Figs 1B, 4B, 7B). Fang relatively straight, curved only near distal end (Figs 1B, 4B, 7B). Opisthosoma elongated, narrow (Figs 1B, 2B), light coloured, with dark, net-like markings on dorsal side (Fig. 1B). Legs slender, long, uniform, except for femur of leg I being thicker than in other legs (Figs 1B, 2B). Measurements of legs: I. 8.94, II. 6.96, III. 8.37, IV. 7.36. Leg spination: I. Fmd 1-1-3ap; Ptpr 1, rt 1; Tbv 2-2-0-2ap, pr 1-0-1-1, rt 1-0-1-1; Mtv 2-0-2, pr 1-0-1ap, rt 1-0-1ap. Leg II. Fmd 1-1-4ap, pr 1, rt 1; Ptpr 1, rt 1; Tbv 2-2-0-2ap, pr 1-1-1, rt 1-1-1; Mtv 2-0-2, pr 1-0-1ap, rt 1-0-1 ap.

Palp (Fig. 5B, F) with retrolateral loop of spermophore wide and occupying about ^1^/_2_ width of bulb (Fig. 5B). Embolus forming about three complete turns, loosely coiled, narrow, flat and dark (Fig. 5B). RTA long, narrowing to distal end, flat, knife-shaped (Fig. 5B, F).

**Female**. Unknown.

##### Distribution.

Known only from the type locality (Fig. 10A).

#### Canama horobi

Taxon classificationAnimaliaAraneaeSalticidae

sp. nov .

12A20141-D49A-5786-89FF-6CB14580056E

https://zoobank.org/EF5FB1AA-CCBA-48FC-B556-EBE326735751

Figs 1C, 2C, 3C, 4C, 5C, 5G, 6A–D, 6F, 7C

##### Material examined.

***Holotype* male** from Indonesia • West Papua, Batanta Island, Valley of Kalijakut River; 545 m a.s.l.; 0°52.51'S, 130°37.98'E; 10 February 2024; coll. Tibor Kovács & Ronnius Sauyai; 2024-13; HNHMAraneae-11516.

***Paratype* male** from Indonesia • West Papua, Batanta Island, Warai Stream valley; between 0°50.41'S, 130°35.29'E and 0°50.98'S, 130°35.29'E; 22–23 February 2019; coll. Előd Kondorosy; HNHMAraneae-11520.

##### Etymology.

The specific name is a patronym, dedicated to Róbert Horváth, Hungarian ornithologist, who initiated and supported Hungarian biodiversity research in Batanta.

##### Diagnosis.

*Canama horobi* can be easily identified by the bulky (Fig. 6A, D), shield-like chelicera (Fig. 1C), with a complex multicuspid teeth (Figs 6B, 6D, 7C). Its palp is similar to those of *C. limagigi* and *C. seltuttensis* in an embolus with many loose coils. The embolus of *C. horobi* differs from *C. limagigi* in having three coils (Fig. 5C vs two in *C. limagigi*; Fig. 5D) and from *C. seltuttensis* by the distally curved RTA (Fig. 5G vs straight then bent RTA of *C. seltuttensis*; Fig. 5F).

##### Description.

Male (holotype). Habitus shown in Figs 1C, 2C. Total length 6.55; carapace length 3.45; width 2.78; abdomen length 3.10; width 1.74; chelicera length 1.51; width 1.57. Prosoma round from dorsal view and darkly coloured, with lighter markings between eyes and at thoracic hump (Figs 1C, 2C). PLE small (Figs 1C, 3C). Chelicera (Figs 4C, 6A–D, 7C) dark brown, stout, wide and shield-like. Chelicerae with two posterior teeth: one small on retromarginal side and a large, wide tooth on promarginal side (Figs 4C, 6A–D, 7C). Paturon with a large multicuspid tooth near base, which bears four tips: one large, pointed tip on retrolateral side of tooth and one large, pointed and two smaller tips on prolateral side (Figs 6A–D, 7C), with one located at base of tooth. Fang with a cusp near distal end (Figs 7C). Fang slightly curved at distal end (Figs 6B, 6C, 7C). Opisthosoma elongated, narrow (Figs 1C, 2C), lighter coloured, with darker markings and scattered orange dots on dorsal side (Fig. 1C). Legs slender; first and second legs thicker and more robust than others (Fig. 1C). Legs I and II darker than others (Figs 1C, 2C). Measurements of legs: I. 7.41, II. 6.51, III. 8.02, IV. 7.01. Leg spination: Leg I. Fmd 0-1-1-3ap; Ptpr 1, rt 1; Tbv 2-1-0-2, pr 1-0-2-1, rt 1-1-1; Mtv 2-2-2ap, pr 1-0-0, rt 1-0-0. Leg II. Fm 0-1-1-4ap; Ptpr 1, rt 1; Tbv 2-2-2ap, pr 1-1-1, rt 1-1-1; Mtv 2-0-2, pr 1-0-1, rt 1–0-1. Palp (Fig. 5C, G) light brown; retrolateral loop of spermophore wide, occupying about ^3^/_4_ of width of bulb (Fig. 5C). Spermophore with a long stem (Fig. 5C). Embolus coiled about three complete turns, narrow, flat (Fig. 5C). RTA long, narrowing until distal end, curved at distal end (Fig. 5 G), anteriorly facing but with curve ventrally facing.

**Female**. Unknown.

##### Distribution.

Known only from the type locality, Batanta Island (Fig. 10B).

#### Canama limagigi sp. nov.

Taxon classificationAnimaliaAraneaeSalticidae

7604ACC2-D984-5403-85F7-BED7D422BC58

https://zoobank.org/35DE9540-CE1F-4EDE-8881-BE6237A7AF63

Figs 1D, 2D, 3D, 4D, 5D, 5H, 6E–H, 7D, 11C, 11D

##### Material examined.

***Holotype* male from** Indonesia • West Papua, Batanta Island, Valley of Warai Stream; 225 m a.s.l.; 0°50.85'S, 130°35.23'E; 21 February 2024; coll. Tibor Kovács; 2024-20; HNHMAraneae-12107.

**Figure 11.**
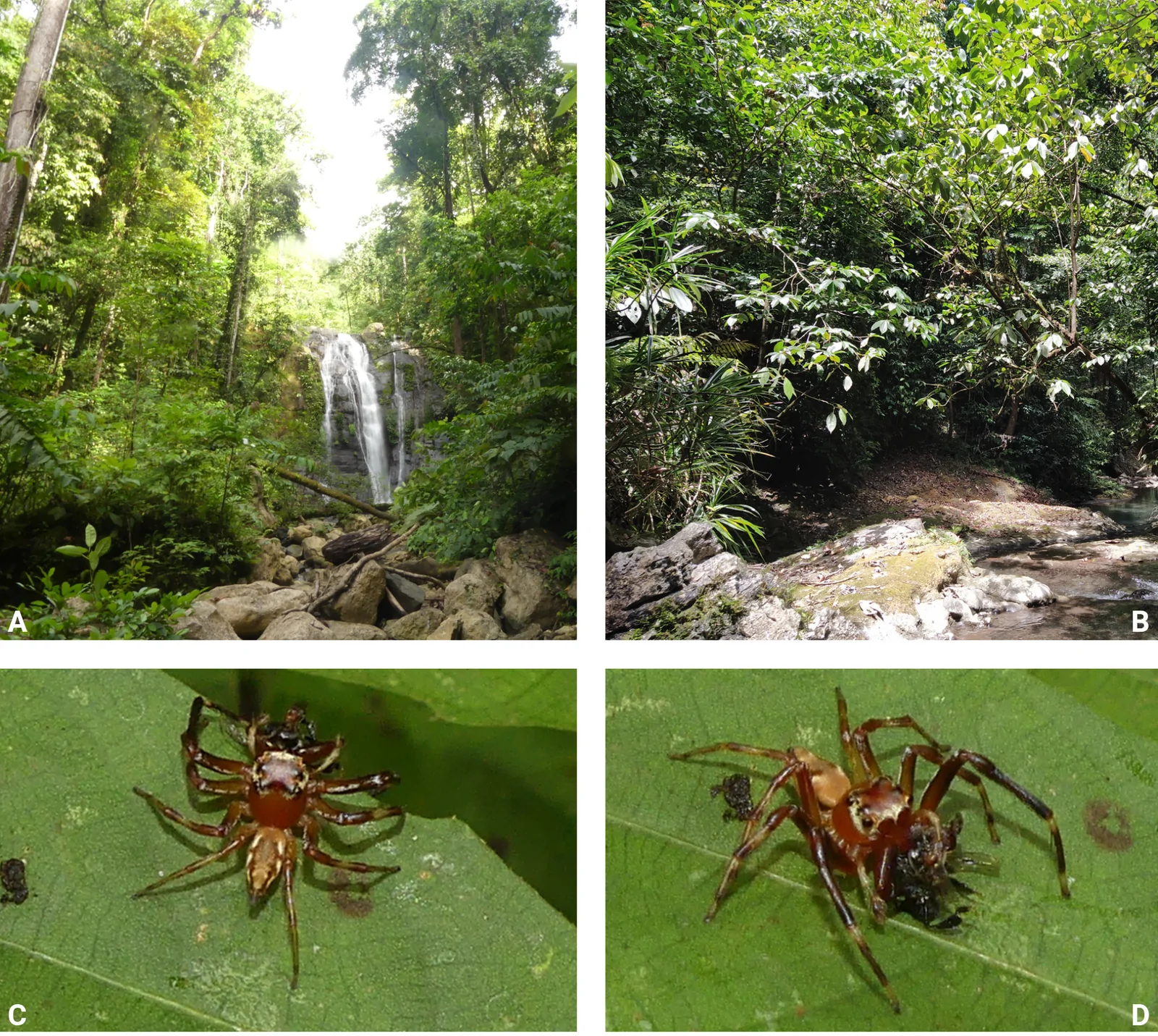
Habitat and living specimen images. **A**. Habitat near Kalijakut River; **B**. Habitat near Warai Stream; **C, D**. *C. limagigi* male paratype from Valley of Waibin River, Batanta.

**Figure 12.**
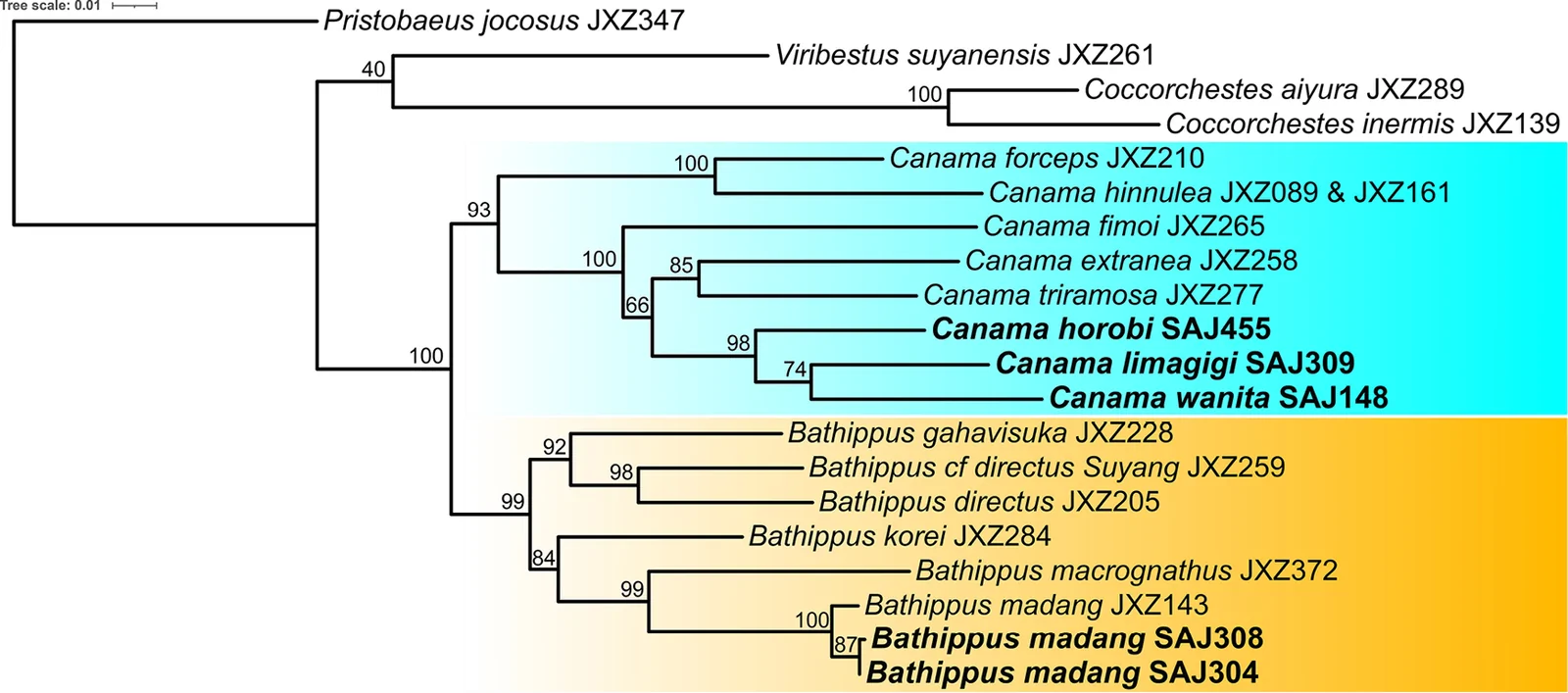
ML tree of the phylogenetic analysis based on the 3 loci. The new sequences generated by this study are highlighted.

***Paratypes*: Batanta Island**: • one male from Valley of Warai Stream; 225 m a.s.l.; 0°50.85'S, 130°35.23'E; 21 February 2023; coll. Tibor Kovács, Péter Juhász; 2023-20; HNHMAraneae-11457.

• 1 male from Valley of Kalijakut River; between 0°53.04'S, 130°38.20'E and 0°52.51'S, 130°37.97'E; 09 February 2024; coll. Tibor Kovács; 2024-11; HNHMAraneae-11466. • 1 male from Valley of Waibin River and right side stream of Waibin River; between 0°49.34'S, 130°45.94'E and 0°50.07'S, 130°45.48'E; 18 February 2024; coll. Tibor Kovács; 2024-17; HNHMAraneae-11929.

##### Etymology.

The specific epithet is a noun in apposition, formed from the Indonesian words ‘*lima*’ (meaning five) and ‘*gigi*’ (meaning teeth) referring to the five teeth on the male chelicerae of the species.

##### Diagnosis.

The palp of *C. limagigi* has a large, bent RTA, as in *C. dorcas* Thorell, 1881, *C. forceps*, and *C. hinnulea* Thorell, 1881, but *C. limagigi* can be differentiated from those species by the narrower embolic coils occupying about half of the bulb’s width (vs occupying more than half of the bulb’s width), and the number of the embolic coils (about two complete turns; Fig. 5D). *Canama limagigi* has a unique number and position of teeth on the chelicerae as well; it has five teeth (Figs 6E–H, 7D): two on the retromargin (one elongated tooth near the fang base and another larger tooth near the base of the paturon) and three on the promargin.

##### Description.

Male (holotype). Habitus shown in Figs 1D, 2D, 4D. Total length 7.73; carapace length 3.75; width 2.84; abdomen length 3.98; width 2.09; chelicerae length 2.11; width 0.92. Prosoma round from dorsal view (Figs 1D, 3D, 4D), light coloured, with dark markings between eyes (Figs 1D, 3D). PLE small (Figs 1D, 4D). Chelicerae (Figs 6E–H, 7D) light brown, relatively short and wide (Fig. 6E–H). Chelicera with five teeth: two large retromargin teeth and three small promargin teeth (Fig. 6F–H). Fang slightly curved on distal end and with a small cusp near base (Figs 6F, 7D). Opisthosoma elongated, narrow (Figs 1D, 2D), light in coloration, with two dark, parallel markings on dorsal side (Fig. 1D). Legs slender: leg I and II more robust and darker coloured (Figs 1D, 2D) than the other legs. Measurements of legs: I. 10.70, II. 8.10, III. 9.63, IV. 8.24. Leg spination: Leg I. Fmd 0-1-1-3ap; Ptpr 1, rt 1; Tbv 2-2-2ap, pr 1-0-1-1, rt 1-0-1-1-1; Mtv 2-0-2, pr 1-0-1ap, rt 1-0-1ap. Leg II. Fmv 0-1-1-4ap; Ptpr 1, rt 1; Tbv 2-2-2ap, pr 1-0-1-1, rt 1-1-1; Mtv 2-0-2, pr 1-0-1ap, rt 1-0-1ap. Palp (Fig. 5D, H) light brown; retrolateral loop of spermophore wide, occupying about ^3^/_4_ of bulb width (Fig. 5D). Embolus coiled, forming two complete turns (Fig. 5D). RTA long, narrowing to distal end, curved at distal end (Fig. 5H), anteriorly facing with curve antero-ventrally facing. Tip of RTA darker than base (Fig. 5H).

**Female**. Unknown.

##### Distribution.

Known from Batanta Island and Birie Island (Fig. 10B).

#### Canama wanita sp. nov.

Taxon classificationAnimaliaAraneaeSalticidae

005D1E02-A86F-57C1-B3FE-CDDE9BB3A779

https://zoobank.org/F4B33711-9D5B-4A11-B40F-A94895911DB9

Figs 8, 9

##### Material examined.

***Holotype* female** from Indonesia • West Papua, Birie Island, near Papua Paradise Eco Resort; 5 m a.s.l.; 0°46.23'S, 130°44.76'E; 26 February 2019; sweeping; coll. Előd Kondorosy; HNHMAraneae-9284.

***Paratypes*: Birie Island**: • 1 female from Papua Paradise Eco Resort and coast; between 5-18 m a.s.l.; 0°46.23'S, 130°44.76'E and 0°46.63'S, 130°44.4'E; 06 February 2025; coll. Tibor Kovács; 2025-2b.; HNHMAraneae-11464. **Batanta Island**: • 1 female from Valley of Wailebet Stream, between 70-290 m a.s.l.; between 0°53.63'S, 130°39.26'E and 0°53.10'S, 130°38.98'E; 14 February 2023; coll. Tibor Kovács, Róbert Horváth, Péter Juhász, Kristian Sauyai, Ronnius Sauyai; 2023-14; HNHMAraneae-11656. • 1 female from Valley of Waibin River; between 00°49.34’S, 130°45.94’E and 00°50.03’S, 130°45.41’E; 05 February 2024; coll. Tibor Kovács; 2024-9; HNHMAraneae-11712. • 1 female from Valley of Warikambon stream, between the War Inkabom and Injonabor waterfalls; 35–145 m a.s.l.; between 0°50.05'S, 130°42.9'E and 0°50.37'S, 130°42.58'E; 10 February 2023; coll. Tibor Kovács, Róbert Horváth, Péter Juhász; 2023-11; HNHMAraneae-11451. **Arefi Island**: • 1 female from Arefi, Mandur; 25 m a.s.l.; 0°47.26'S, 130°42.36'E; 10 February 2019; coll. Előd Kondorosy; HNHMAraneae-11506.

##### Etymology.

The species is a noun in apposition, derived from the Indonesian word *wanita* (meaning woman, female), referring to the fact that the species is known only from females.

##### Diagnosis.

The female genitalia of *C. wanita* can be differentiated from females of all other *Canama* species by the circularly curved copulatory ducts, visible as two half circles through the cuticle (Fig. 9A, B), and by the wide median septum.

**Male**. Unknown.

**Female**. Female (paratype). Habitus shown in Fig. 8. Total length (holotype) 6.75; carapace length 3.05; width 2.40; abdomen length 3.70; width 2.20; chelicerae length 0.96; width 0.73. Prosoma round from dorsal view (Fig. 8A), light yellow, with darker markings between eye-field (Fig. 8A). Chelicerae yellow, with two small, unident, promargin teeth and two separate retromarginal teeth (Fig. 8E). Opisthosoma elongated, narrow (Fig. 8), with two dark, parallel markings on dorsal side. (This marking probably covers the whole abdomen, as a paratype with a light dorsal longitudinal stripe on a dark background also has been captured.) Legs slender, rather uniform in appearance. Measurements of legs (HNHM11541): I. 4.69, II. 4.71, III. 5.32, IV. 4.67. Leg spination: Leg I. Fmd 0-1-1-4ap; Ptpr 1, rt 1; Tbv 2-2-2ap, pr 1-0-1, rt 1-0-1, d 1-2-0; Mtv 2-0-2, pr 1-0-1ap, rt 1-0-1ap.

Leg II. Fmd 0-1-1-5ap; Ptpr 1, rt 1; Tbv 2-2-2ap, pr 1-0-1, rt 1-0-1, d 1-2-0; Mtv 2-0-2, pr 1-0-1ap, rt 1-0-1ap Epigyne (Fig. 9) with small epigynal window, median septum short and wide (Fig. 9A–C), about size of epigynal window, opening to copulatory duct located at anterior end of window. Copulatory duct wide, turned backwards towards epigastric fold (Fig. 9A–C); spermathecae medium-sized (Fig. 9D–F).

##### Distribution.

Known from Arefi, Batanta and Birie islands (Fig. 10).

##### Remarks.

The holotype female was sequenced, using the COI gene, to test for their conspecificity with the males of species on Batanta Island. However, the genetic distances between the holotype of *C. wanita* and *C. limagigi* was found to be 8.346%, and for *C. horobi* it was 9.621%. These distances are too great to assign the female *C. wanita* to either of these species. We acknowledge that this might be the unknown female of either *C. dorcas* or *C. forceps*.

### Molecular phylogenetic analysis

Both maximum-likelihood (IQTREE and PhylML) and Bayesian interference (MrBayes) analyses resulted trees of the same topology (Fig. 12, Suppl. material 1: fig. S1). All three new *Canama* species were deeply placed in *Canama* as a monophyletic group (*C. wanita* and *C. limagigi* are sister to each other) and show a close relationship to a group consisting of *C. fimoi*, *C. extranea*, and *C. triramosa*. Despite the smaller overlap of COI sequences (the barcode region and the extended region ~300 nts) in our final dataset, it most likely did not significantly affect our results of the ML and BI analyses. However, the heterogeneity of our dataset may complicate comparisons between historical and our newly generated data. Thus, extra effort should be put amplifying a longer COI fragment, covering both the barcoding and extended regions of the gene.

## Discussion

The clear boundaries of *Canama* and *Bathippus* have already been settled (Zhang and Maddison 2012, 2015), but there is still much more diversity to discover in these clades. Our results are consistent with the results of Zhang and Maddison (2013, 2015), and we were able to examine two species described over a century ago. Our knowledge on these species is still thin: six of the nine *Canama* species and 25 of the 30 *Bathippus* species are known from only one or two papers, and often without clear illustrations (World Spider Catalog 2026). The character systems (i.e. chelicera, teeth, and palps) vary widely. Clear diagnoses and testable morphological characters are needed for the placement of species, as the enlarged chelicerae are not sufficient to place species in these genera (Zhang and Maddison 2012).

## Supplementary Material

XML Treatment for Canama

XML Treatment for Canama dentiferellus

XML Treatment for Canama dilanians

XML Treatment for Canama seltuttensis

XML Treatment for Canama horobi

XML Treatment for Canama limagigi

XML Treatment for Canama wanita

## Additional information

### Artificial Intelligence (AI) use

The authors accept full responsibility for the content of the manuscript, including the disclosure of any use of AI.

No AI tools were used in the preparation of this manuscript.

### Author ORCIDs

H. Takács-Vágó https://orcid.org/0009-0003-7837-1979

Á.S. Mayer https://orcid.org/0009-0009-0989-5078

K. Szabó https://orcid.org/0000-0002-5110-8279

T. Kovács https://orcid.org/0000-0003-4577-0394

T. Szűts https://orcid.org/0000-0001-8954-0641

### Data availability

All of the data that support the findings of this study are available in the main text or Supplementary Information.

## Supplementary materials

Supplementary material 1 Supplementary data Data type docx Explanation note **fig. S1**. MrBayes consensus tree of the phylogenetic analysis based on the 3 loci. FINAL ALIGNMENT (3319). MODEL SELECTION. https://binary.pensoft.net/file/1795799This dataset is made available under the Open Database License (http://opendatacommons.org/licenses/odbl/1.0/). The Open Database License (ODbL) is a license agreement intended to allow users to freely share, modify, and use this Dataset while maintaining this same freedom for others, provided that the original source and author(s) are credited. Hunor Takács-Vágó, Ádám Sándor Mayer, Krisztián Szabó, Tibor Kovács, Tamás Szűts
