# Supplementary material for: First chance to see: New jumping-spider species from Batanta, Indonesia (Araneae, Salticidae, Euophryini)
Source: Zookeys. 2026 Oct 1;1293:241–63. doi: 10.3897/zookeys.1293.190325 (PMC13650590; doi:10.3897/zookeys.1293.190325)
Supplement: Supplementary file 1 [file zookeys-1293-241_article-190325__-s001.docx]

Table of contents

[Figure S1.	2](#_Toc236412981)

[FINAL ALIGNMENT (3319)	3](#_Toc236412982)

[MODEL SELECTION	23](#_Toc236412983)

# Figure S1.

**Figure S1. MrBayes consensus tree of the phylogenetic analysis based on the 3 loci.** The new sequences generated by this study are highlighted.

# FINAL ALIGNMENT (3319)

>Pristobaeus jocosus JXZ347

TTAATAGTAAAATCTGCTCAATGA----CAT-TTCMATAGCCGCAATA-AGTGCTAAGGTAGCATAATCAT-TTGTCTTTTAAATAAAGACTAGAACAAAAGATTTAACATTTCAAATTACCTATTCAATTTTTTTATTAAAATTATTTTAAATGTAAAAAAACATTTATACAAAAAAAAGACGATAAGACCCTATTGAACTTAACTTTAGTTAAACTGGGGTAGTAAATTAAATATTATTTTAATATAAATGTATAAAAAAATATCCATTAAAATTGAATTATTAAGTTACCATAGGGATAACAGCGCAATTATATTTTAAAGCTCTTATTTAAAAAATAGATTACGACCTCGATGTTGAATTAATAACC---TATTTTATCAAAGCAAATTTTAGAA-AAGGAAGTCTGTTCGACTTTTTAAAAATTACATGATTTGAGTTCAGACCGGTGCAAGCCAGGTCGGTTTCCATCTTTTAAATATAATTATACAGTACGAAAGGACCTATAAATTTAATTTAATTTAAAA-TTTGGCAGATAAATGCATTAGAATTAGAATCTAATTAACTATAAATTATTTTTTCAATAAATTTCATTATAATTACTATCAGAATTTTAGTAAGAGTAGCATTTTATACTATCTTAGAACGTAAATTGTTAGGATATATTCAAATACGAAAAGGTCCTAATAAAGTGGGATTTATAGGCATCTTACAACCATTCAGAGACGCCATTAAATTATTCAATAAAAATTTAATTACAAACGAAAATATAAATATAATTCTATCATTTATCTCTCCAGCCTTATCATTATTAATCAGAATTTTATTA--------------------------------------------------------------------------------------------------------------------------------------------------------------------------------------------------------------------------------CCGGCGGCAAGTCCCCCTGACCGGGGCCTGTCCGTAGCGGGTGCGAGGCCCATAGCCGCCGGAGCCGTCCTCGAATGCGATCTCCTGGGAGTCGGGTTGCTTGGGAGTGCAGCCCCAATCGGGTGGTAAACTCCACCTAAGGCTAAATACCGCCGTGAGACCGATAGCAAACAAGTACCGTGAGGGAAAGTTGCAAAGAACTTTGAAGAGAGAGTTCAAGAGTACGTGAAACTGCTCAGAGGTAAACGGGTGGGCCCTCGAAATCCTGTGGCGAGAGGATTCAGTCTGTGCGGGCGGACGCCT--GCGGAGGAAGATC----GGAAGGGCGACCGC-TGA-CGCGGGCCGTCCTCCGTGG-CG---CCTTTCGCCCCCGCAGACGCATTTGTCTCTCGTCCCGAGGACGCTGCAGCCGGTCTGGAAG-CGCAAGCGCGACGGCCTGAAGGCCGGGCGAC-GGCAGGTGGCCGGTGGCTC-GCCTCG-TGCGGGTCGTCGGTTTTTATAGCCGGCCCGGCAGTGGCTCGTCTCCAGACCGTGGTGT-CGCGAGTCCCCTTCCGGGGACTCCTCGTCTCCACCCC----AGAGTC-GCGGGACAGGGCGCGGCCAGGG-CCCC-GCGTCGCGA----CCGTCGCACTTCCACGGAGTGGACGGAAGGGCTCGGGGCCCGC--ACCGCCGCGACGGA--CCCGCGGGGGATCGGAGGCTGGCAGCGAGTAGGTCGGTCACCCACCCGACCCGTCTTGAAACACGGACCAAGGAGTCTAACATGTGCGCGAGTCAATGGGTCTTGAACAGGCCCATGGGCGCAATGAAAGCGAAAGG-CCGCC-GAGCGCG-G---TCCGAGTGGGGATCCCGGGGGCTCGT-CCCTCGGGCGCACCCGCGACCCGTCCTATCCGGCTCGCCGGTTGGGCGGAGTCTGAGCGTACACGTTGGGACCCGAAAGATGGTGAACTATGCCCGGACAGGACGAGGCCAGAGGAAACTCTGGTGGAGGTCCGCAGCGGTTCTGACGTGCAAATCGATCGTCAGATCTGGGTCTAGGGGC-----------------------------------------------------------------------------------------------------------------------------------------------------------------------------------------------------------------------TTTTCCTCGAATAAATAATTTGAGATTTTGATTGCTTCCACCATCTTTAATATTATTATTTATATCTTCTATAGTTGAGATAGGGGTGGGTGCTGGATGGACTGTTTATCCTCCATTAGCTTCTGTTATTGGTCATAATGGTAGTTCTGTGGATTTTGCTATTTTTTCTTTACACTTAGCTGGAGCTTCTTCAATTATAGGAGCTGTAAATTTTATCTCTACTATTATTAATATACGTTCTATTGGGATATCTATAGATAAATTTTCTTTATTTGTATGATCTGTTTTAATTACTGCTATTTTATTATTATTGTCTTTACCGGTTTTAGCTGGTGCTATTACTATATTATTGACTGATCGAAATTTTAATACTTCTTTTTTTGACCCTGCTGGGGGTGGGGATCCTATTTTATTTCAACATTTATTTTGATTTTTTGGTCATCCTGAGGTTTATATTCTTATTTTACCAGGTTTTGGTATTGTATCTCATGTGATTAGATCTTCGGTTGGTAAACGTGAACCTTTTGGTTCTTTAGGAATAATTTATGCTATATTAGGAATTGGTGGAATGGGATTTGTAGTATGAGCTCATCATATATTTTCAGTTGGAATAGATGTTGATACTCGGGCTTATTTTACAGCTGCTACCATAATTATTGCAGTTCCTACTGGAATTAAAGTATTTAGATGAATAGCTACATTATATGGTTCTTATTTTAAGATAAGAACTCCTTTATTGTGATCTATTGGGTTTGTATTTTTATTTACTTTAGGTGGAATTACTGGGGTAGTTTTGTCTAATTCTTCTTTAGATATTATTCTTCATGATACTTATTATGTAGTTGCCCATTTTCATTATGTATTGAGAATAGGAGCTGTTTTTGCTATTATAGCTGGAATTACCTATTGATTTCCTTTATTTTTTGGTACAATTTTGAATGAGAAAAAAACTAAGTTGCAATTTTTTACTATATTTTTAG--------

>Coccorchestes aiyura JXZ289

---------------------TGA----TAA-TTAAATAGCCGCAATT-CGTGCTAAGGTAGCATAATAAT-TTGTCTTTTAAATAAAGACCAGAACAAAAGATTCCACATTTTAGCTATTCTATTTCTTATATAAATTAAAATTATACAAAATGTAAAAAAACATTTATTTAAAAGAAAGACGACAAGACCCTATTAAACTTAACTTCAGTTAAACTGGGGCAGTAAATTAATTATTATTTTAATTATATTAAATTACCAATAATCTAAAA--CATTAATAAATCAGTTACTATAGGGATAACAGCGCAATTATATTTTAAAGTACTCATTTAAAAAATAGATTACGACCTCGATGTTGAATTAATTACC---TAATTA----AAGCAAAAATTAAAT-AAGGAAGTCTGTTCGACTTTTTAAAAATTACATGATTTGAGTTCAGACCGGTGTAAGCCAGGTCGGTTTCAATCTACTAAAAACTTTCTTACAGTACGAAAGGGCCTAAGAATATTTTAT-----ATAA-ATTGGCAGATAAATGCATTAGAATTAGAATCTAATAAAACTAAAATTCTCTTCTCTTTAATATATATATTAATCTTAATTAGAATTTTATTAAGAGTAGCTTTCTACACTATTTTAGAACGAAAAATTTTAAGATATATTCAAATTCGAAAAGGACCTAACAAAGTAGGTATTATAGGAATTCTACAACCCTTTAGAGACGCAATAAAACTTTTTAATAAAAACCTTATTTTATCTGAAAACAGAAATTCCATTCTACTATATATTATACCAGCATTATCCTTATTCATCAGCATAATACTTATTCCTATTTTATCTTATAATTCAATATCATTATATGATAATAAACACAGAATTCTACTATTTTTCATTTTATCAAGAATTTCAGTTTATTCAACTCTAATAATTGGATGATCAGCTAA---------------------------------------------------------------------ATGTGGCGTTTAGGAGTGAGCGCGTCGGGCGCGGGGAGGGCGGCAAGTCCCCCTGACCGGGGCCTGTCCGTAGCGGGTGCGAGGCCCATAGCCGCCCGCCCCGC-CCCGAACGCGATCTCCTGGGAGTCGGGTTGCTTGGGAGTGCAGCCCCAATCGGGTGGTAAACTCCACCTAAGGCTAAATACCGCCGTGAGACCGATAGCAAACAAGTACCGTGAGGGAAAGTTGCAAAGAACTTTGAAGAGAGAGTTCAAGAGTACGTGAAACTGCTCAGAGGTAAACGGGTGGGCCCTCGAAATCCTGTGGCGGGAGGATTCAGTCTGGGCCGAGGGGCGGCG--CCGGGGAAAGCGTGT--GGAAGGGCATCCGCCGAG-CGCGGGCCGTTCCCGGCGC-CGT-CGATCCCCCAGTCCCAGACGCATTTGACTCCCGTCCCGAGGACGCTGCGGCCGGTCCGGAAGTTGCAAGCGCGACGGCCTGGAGGCCGGGAGAC-GGCAGGTGGCCGGTGGCCC-GCCGAGCTGCGGGTCGCCGGTTGT--TAGCCTTCCCGGCGGTGGCACGTCTCCGGACCGAGGTGCGAGCGGGTCCCCGCACGGGGACCCACCGTCTCCGCTCCCCACCGAAGC-GCGGGACAGACCGCGGCCCGGGGCCCC-TGGATCCGA----CCGGCGCACCACCACGCGGTGGCCGGGAGGGCCGGGGTCCCACAGACCGCCGCGGCGGA--CCCGCGGGGGACCGGAGGCCGGCAGCGAGTAGGTCGGTCACCCACCCGACCCGTCTTGAAACACGGACCAAGGAGTCTGACATGTGCGCGAGTCGACGGGTCTTGGACAGGCCCGTGGGCGCAATGAAAGCGAAGGG-CCGCCTGGGCGCG-G---CCTGAGCGGGGATCCCCCGTCCCCGA-GGCGGGGGCGCACCCGCGACCCGTCCCGTCCGGCTCGCCGGTGGGGCGGAGTCCGAGCGCACGCGTCGGGACCCGAAAGATGGTGAGCTATGCCCGGGCAGGACGAAGCCAGAGGAAACTCTGGTGGAGGTCCGCAGCGGTTCTGACGTGCAAATCGATCGTCAGATCCGGGTATAGGGG-------------------------------------------------------------------------------------------------------------------------------------------------------------------------------------------------------------------------TTTCCTCGTATAAATAATTTAAGTTTTTGATTATTACCACCTTCTTTAATACTATTATTTATTTCTTCTATAGTTGAAATAGGAGTGGGAGCAGGGTGAACTGTATATCCACCATTAGCTTCTGTTATAGGTCATAGTGGAAGTTCTGTTGATTTTGCTATTTTTTCTTTACATTTGGCTGGTGCTTCATCTATTATAGGTGCTATTAATTTTATTTCAACTATTTTAAATATACGCTCTATTGGGATATCTATAGATAAAATTTCTTTATTTGTATGATCTGTTATAATTACAGCTATTTTATTATTATTGTCTTTGCCAGTATTAGCTGGAGCTATTACTATATTATTAACGGATCGTAATTTTAATACTTCTTTTTTTGATCCTGCAGGAGGGGGGGATCCTATTTTATTTCAACATTTATTTTGATTTTTTGGTCATCCAGAGGTTTATATTTTAATTTTACCTGGATTTGGAATTGTATCTCATGTTATTAGAGCTTCAGTTGGAAAACGTGAACCTTTTGGTTCTTTAGGAATAATTTATGCAATAGTAGGTATTGGTGGAATAGGATTTGTTGTATGAGCACATCATATATTTTCAGTGGGAATGGATGTTGATACTCGGGCATATTTTACAGCGGCAACTATAATTATTGCAGTACCTACTGGTATTAAGGTTTTTAGATGAATAGCTACATTATATGGTTCGTATTTTAAAATTGATACACCTTTAATATGAAGTATTGGTTTTGTTTTCTTATTTACTTTAGGAGGTATTACGGGAGTAGTTTTATCTAATTCTTCTCTCGATATTATTTTACATGATACCTACTATGTAGTTGCACATTTTCATTATGTATTGAGTATAGGAGCTGTATTTGCAATTTTAA-----------------------------------------------------------------------------------------------

>Coccorchestes inermis JXZ139

--------------------------------TTAAATAGCCGCAATT-TGTGCTAAGGTAGCATAATAAT-TTGTCTTTTAAATAAGGACCAGAACAAAAGATTTCACATTTTAATTATTTTATTTCCTATAAAAACCCAAATTACATAAAATGTAAAAATTCATTTATTAAAAAGAGAGACGATAAGACCCTATTAAACTTAACTTTAGTTAAACTGGGGCAGTAAATTAATTAATATTTTAATTACTTTAAATAAATATTTATCTAATA-ATATTAATAATCCAGTTACTATAGGGATAACAGCGCAATTATATTTTAAAGTACTTATTCTAAAAATAGATTACGACCTCGATGTTGAATTAATTACC---TAATTA----AAGCAAAAATTAAAT-AAGGAAGTCTGTTCGACTTTTTAAAAATTACATGATTTGAGTTCAGACCGGTATAAGCCAGGTCGGTTTCAATCTTCTAAAAACTTTCTTACAGTACGAAAGGACCCAAGAATATTATAT-----TTAA-ATTGGCAGATAAATGCATTAGAATTAGAATCTAATAAAACTAAAATTCTTTTCTCCTTTATATATATCCTAATTCTAATTAGAATATTATTAAGAGTAGCTTTCTATACTATTTTAGAACGAAAAATCTTAAGATATATTCAGATTCGAAAGGGACCTAACAAAGTAGGTATTATAGGTATATTACAACCTTTTAGAGATGCAATAAAACTTTTTAATAAAAACACTTTATTATCTGAAAATAGAAATTTTATCCTTCTATATATTATACCAGCAATATCACTATTTATTAGAATAATACTCATTCCTATTTTATCTTATAATTCCATATCCTTATATGATAATAAACATAGAATTCTCTTATTCTTCATTTTATCAAGAATTTCAGTTTATTCAATTCTAATAA--------------------------CGGCGAGCGAAAAGGGATGAGCCCAGCGCCGAATCCCCGACGGTCGCCCGTCGCGGGAAATGTGGCGTTTAGGAGTGAGCGCGCCGGGCGCGGCGGAGGCGGCAAGTCCCCCTGACCGGGGCCTGTCCGTAGCGGGTGCGAGGCCCATAGCCGCCCCCTCCGT-CCCGGACGCGATCTCCTGGGAGTCGGGTTGCTTGGGAGTGCAGCCCCAATCGGGTGGTAAACTCCACCTAAGGCTAAATACCGCCGTGAGACCGATAGCAAACAAGTACCGTGAGGGAAAGTTGCAAAGAACTTTGAAGAGAGAGTTCAAGAGTACGTGAAACTGCTCAGAGGTAAACGGGTGGGCCCTCGAAATCCTGTGGCGAGAGGATTCAGTCTGGGCCGAGGGGCGGCG--CCGGGGAAAGCGTT---GGAAGGGCTTCCGCCGGG-CGCGGGCCGTTCCCGGCGT-CGCGAGATCCCCCAGTCCCAGACGCATTTGACTCTCGTCCCGAGGACGCTGCGGCCGGTCCGGAAGTTGCAAGCGCGACGGCCTGGAGGCCGGGAGAC-GGCAGGTGGCCGGTGGCCC-GCCGAGTTGCGGGTCGCCGGTTGT--TAGCCATCCCGGCGGTGGCACGTCTCCGGACCGAGGTGTGGGCGGGTCCCGTCAC-GGGACCCCACGTCTCCGCTCCCCACCGAAGC-GCGGGACAGACCGCGGCCCGGGGCCCCGTTTTTCCGA----CCGGCGCACCACCACGCGGTGGCCGGGAGGACCGGGGTCCCACGAGCCGCCGCGGCGGA--CCCGCGGGGGACCGGAGGCCGGCAGCGAGTAGGTCGGTCACCCACCTGACCCGTCTTGAAACACGGACCAAGGAGTYTGACATGTGCGCGAGTCGACGGGTYTTGGACAGGCCCGTGGGCGCAATGAAAGCGAAGGG-CCGCCTGGGCGCG-G---CCTGAGCGGGGATCCCCCGTCCCCGA-GGCGGGGGCGCACCCGCGACCCGTCCCGTCCGGCTCGCCGGTGGGGCGGAGTCCGAGCGCACGCGTCGGGACCCGAAAGATGGTGAGCTATGCCCGGGCAGGACGAAGCCAGAGGAAACTCTGGTGGAGGT-----------------------------------------------------------------------------------------------------------------------------------------------------------------------------------------------------------------------------------------------------------------------------------------AATTTGAGTTTTTGATTATTGCCTCCTTCCTTAATGTTATTGTTTATTTCTTCAATAGTTGAGATAGGAGTGGGAGCAGGGTGAACTGTATATCCACCATTAGCGTCTATTATAGGACATAATGGAAGTTCTGTTGATTTTGCTATTTTTTCTTTACATTTGGCTGGTGCCTCATCTATTATAGGTGCCATTAATTTTATTTCAACTATTTTAAATATACGTTCTATTGGAATATCTATAGATAAAATTTCTTTATTWGTATGATCCGTTATAATTACAGCTATTTTATTATTATTGTCTTTACCAGTGTTAGCTGGTGCAATTACTATATTATTGACGGATCGTAATTTTAATACTTCTTTTTTTGACCCTGCAGGGGGGGGAGATCCTATTTTATTTCAACATTTATTTTGATTTTTTGGTCATCCGGAGGTTTATATTTTGATTTTACCAGGGTTTGGAATTGTGTCTCATGTTATTAGATCTTCAGTTGGGAAACGAGAACCATTTGGTTCTTTAGGGATAATTTATGCAATAGTAGGAATTGGTGGAATAGGATTTGTTGTATGAGCACACCATATATTTTCAGTGGGAATAGATGTTGATACTCGAGCGTATTTTACGGCAGCTACTATAATTATTGCAATTCCTACTGGTATTAGGGTATTTAGATGAATAGCTACATTATATGGATCCTATTTTAGGWTTGATACACCTTTAATGTGATGTATTGGTTTTGTTTTCTTATTTACTTTAGGGGGTATTACAGGAGTAATTTTATCTAATTCTTCTCTTGATATTATTTTACATGATACTTATTATGTGGTTGCCCATTTTCATTATGTATTAAGAATAGGTGCTGTATTTGCAATTTTGAGAGGAATCACTTA----------------------------------------------------------------------------------

>Viribestus suyanensis JXZ261

----------------------------ATATTTAAATAGCCGCAATT-TGTGCTAAGGTAGCATAATCAT-TTGTCTTTTAAATGAAGACTAGAACAAAAGACTTAACATTTTAATTTATTTATTAAATATAAATATTCACATTTTTTTAAATGTAAAAAAACATTTATTTTAAAGAAAGACGATAAGACCCTATTGAACTTAACTTAAGTTAAACTGGGGCAGTAAATTAATTATTATTTTAATAAAAATAAACAATTTATTATCTAATA-TAATTAATTATTCAGTTACCATAGGGATAACAGCGCAATTAGCTTTTAAAGCTCTAATTTAAAAGCTAGATTACGACCTCGATGTTGAATTAATAACC---TAACTA----AAGCAACTTTTAGGA-AAGGAAGTCTGTTCGACTTTTAAATAATTACATGATTTGAGTTCAGACCGGTGTAAGCCAGGTCGGCTTCAATCTTCTTAAAACTCTTTTACAGTACGAAAGGACCTAAAAATATAATTTAATTAATTT-ATTGGCAGATAAATGCATTAGAATTAGAATCTAATAAAACTAAAATATTATTTTCCATAAATTATATCTTAATTTTAGTAAGTATCTTAATTAGTGTGGCTTTTTATACTATTTTGGAGCGAAAAATTTTAGGTTATATCCAAATTCGTAAAGGCCCTAATAAAGTAGGAATTCTAGGAATTTTACAACCATTTAGAGATGCTTTAAAATTATTTAATAAAAATCTATTATCTTCTGAAAATATAAACTATATTTTATCTTATATAACTCCAGCTATATCTTTATTTATTAGAATAATATTAATCCCTATTATTATATATAATAATATATCATTATACGATAATAAACACAATATTCTACTTTTTTTTATCCTATCAAGAATAGCCGTATACTCAATCTTATTAATTGGTTGATCAGCCAA-------------------------------------------------------------------------------TAGGAGTGAGCGCGTCGGGTGCGGAGAAGGCGGCAAGTCCCCCTGACCGGGGCCTGTCCGTAGCGGGTGCGAGGCCCATAGCCGCCTCTTCCGC-CTCGAACGCGATCTCCTGGGAGTCGGGTTGCTTGGGAGTGCAGCCCTAATCGGGTGGTAAACTCCACCTAAGGCTAAATACCGCCGTGAGACCGATAGCAAACAAGTACCGTGAGGGAAAGTTGCAAAGAACTTTGAAGAGAGAGTTCAAGAGTACGTGAAACTGCTCAGAGGTAAACGGGTGGGCCCTCGAAATCCTGTGGCGAGAGGATTCAGTCTGGATCGCGCGACGAC---CGGGCGGAAGAATCCG-AGCAGGGCTTCCGCGTCG-CGCGGGCCGTCCGCTCGGT-CG---ACTTTGCGCGACCCAGACGCATTTGTCTCTCGTCCCGAGGACGCCGCAGCCGGTTCGGGAGTTGCAAGCGCGACGGCCTGGAGGCCGGGACGA-CGCAGGTGGCCGGTGGCCC-GCTTAG--GCGGGTCGTCGGTTGT--TAGCGGTCCCGGCAGTGGCTCGTCCCCGGACCGAGGTGT-CGCGAGTCCCTTTGC-GGGATTCGCCGTCTCCACCCC----AGAGTCAGCCGGACAGGGCGCGGAATGGA-GCCC-CGTCGTCGC----CCGTCGCACTTCCACGGAGTGGGCGGGCGGGGCGGGGAGCCGC--ACGGCCGCGACGGA--CCGGCGGGGGACCGGAGGCTTGCGGCGAGTAGGTCGGTCACCCACCCGACCCGTCTTGAAACACGGACCA-----------------------------------------------------------------------------------------------------------------------------------------------------------------------------------------------------------------------------------------------------------------------------------------------------------------------------------------------------------------------------------------------------------------------------------------------------------------------------------------------------------------------------TTTTCCTCGAATAAATAATTTAAGTTTTTGATTATTACCTCCTTCTTTAANNNNNTTATTTATTTCTTCTATAGCGGAAATGGGAGTTGGTNCAGGATGAACTGTTTATCCTCCTTTAGCTTCTATTGTAGGTCATAATGGAAGATCAGTTGATTTTGCTATTTTTTCTTTACATTTAGCTGGGGCTTCTTCTATTATAGGTGCTATTAATTTTATTTCAACTGTTATTAATATACGATCTAGTACAATATCTATAGATAAAATTTCTTTATTTGTATGATCAGTAGTAATTACAGCTGTGTTATTATTATTATCTTTACCTGTTTTAGCAGGTGCTATTACTATATTATTAACAGATCGAAATTTTAATACTTCTTTTTTTGATCCAGCTGGAGGTGGTGATCCTATTTTGTTTCAACATTTATTTTGATTTTTCGGACATCCTGAAGTTTATATTTTAATTTTACCAGGATTTGGTATTGTTTCTCATGTTATTAGAGCTTCAGTAGGAAAGCGTGAACCATTTGGCTCATTAGGGATAATTTATGCAATAATTGGAATTGGAGGAATAGGATTTGTGGTATGAGCTCATCATATATTTTCTGTAGGAATGGATGTTGATACTCGCGCATATTTTACTGCCGCTACTATAATTATTGCTGTTCCAACTGGAATTAAGGTGTTTAGATGAATAGCTACCTTGTATGGATCTTATTTTAAAATTGATACTCCTCTTATATGAAGTATTGGTTTTGTTTTTTTATTTACTTTAGGGGGTATTACAGGGGTAGTTTTATCAAATTCTTCTTTAGATATTATTTTACATGATACCTATTATGTTGTTGCTCATTTTCATTATGTCTTAAGAATGGGAGCTGTATTTGCTATCTTAGCTGGAATT---------------------------------------------------------------------------------------

>Bathippus cf. directus Suyang JXZ259

---------------------TGA----TTA-TTAAATAGCCGCAATT-AGTGCTAAGGTAGCATAATCAT-TTGYCTTTTAAATGAAGACCAGAACAAAAGATTTAACATTTTAATTTATTTATTTAAATATTTAATTAAATTTACCTAAAAAGTAAAAAGGCTTTTCTAAAAAAGAAAGACGACAAGACCCTATCGAACTTAACTTAAGTTTAACTGGGGTAGTAAATTAATTATTATTTTATAATTAAACTATTATTCAAATATCCTTA--AATTGATAATCCAGTTACCGTAGGGATAACAGCGCAATTAAATTTTAAAGTTCTCATTTAAAATATAGATTACGACCTCGATGTTGAATTAATAACC---TATTTC----AAGCAAATTTCAAAA-AAGGAAGTCTGTTCGACTTTTAATAAATTACATGATTTGAGTTCAGACCGGTATAAGCCAGGTCGGTTTCAATCTTCTAAAAACTCTTTTACAGTACGAAAGGACCTAAAAATATAATTTAATTAACTA-ATTGGCAGATTAATGCATTAGAATTAGAATCTAATTAAAATTATATTTCCTTTATATTAAATTATATTCTAATTTTAATTAGAATTTTAATCAGAGTAGCTTTCTACACTATTTTAGAACGAAAAGTTCTAGGATATATACAATTACGAAAAGGACCTAATAAAGTAGGAATAATAGGAATTTTACAGCCTTTTAGAGATGCCTTAAAACTTTTTAATAAAAATTTATTATCTTCAGAAAATATAAATTTCATTCTATCTTACATAACGCCCGCTATATCATTAATAATTAGAATATTATTGATCCCTATTATTCTATATAATAATATATCACTATATGATAATAAACATAGCATCTTATTATTTTTTATCTTATCTAGTATCTCTGTATATTCAATTTTATTAATTGGATGATCCGCCAA-------------------------------------------------------TCTGGTCGCGGGAAATGTGGCGTTTAGGAGTGAGCGTGTCGAGTCCGGAGCCGGCGGCAAGTCCCCCTGACCGGGGCCTGTCCGAAGCGGGTGCGAGGCCCATAGCCGCCGGTCCCGT-CTTGAATGCGATCTCCTTGGAGTCGGGTTGCTTGGGAGTGCAGCCCTAATCGGGTGGTAAACTCCACCTAAGGCTAAATACCGCCGTGAGACCGATAGCAAACAAGTACCGTGAGGGAAAGTTGCAAAGAACTTTGAAGAGAGAGTTCAAGAGTACGTGAAACTGCTCAGAGGTAAACGGGTGGGCCCTCGAAATCCTGTGGCGAGAGGATTCAGTCTTGAGCGGAGGACTTC---CCGGGGGAAGAAACCG-AGCAGGGCGTCCGCGTCGTCGCGGGCCGTCCTCCGGGT-CG---ACCTCAACCGCCAGAGACGCATTTGACTCTCGCCCCGAGGACGCTGCAGCCGGTCTGGAAGTTGCCAGCGTGACGGCCTGAAGGCCGGGAAACAGGCAGGTGGCCGGTGGCTC-GCCGCG-TGCGGGTCGTCGGTTGT--TAGCCGTCCCGGCAGTGGCTCGTCTCCAGACCGAGGTGT-CGCGAGTCCCTTCG--GGGACTCCTCGTCTCCACCCC----AGAGTC-GCGGGACAGGGCACGGAACGGG-TCTT-CGCCGTCAA----CCTCCGCACTGGCACGGAGTGGGCGGAAGACACGGAGCCCCGC--ACCGCCGTGACGGA--CCCGCGGGGGATCGGAGGCTGGCAGCGAGTAGGTCGGTCACCCACCCGACCCGTCTTGAAACACGGACCAAGGAGTCTAACATGTGCGCGAGTCAGTGGGTCTTGAACAGGCCCACGGGCGCAATGAAAGCGAAAGG-CCGCC-GAGCGCG-G---CCCGAGTGGGGATCCCCGCTCTTAGCCGGGCGGGGCGCACCCACGACCCGTCCTATCCGGCTCGCCGGTTGGGCGGAGTCTGAGCGTACACGTTGGGACCCGAAAGATGGTGAACTATGCCCGGACAGGACGAGGCCAGAGGAAACTCTGGTGGAGGTCCGCAGCGGTTCTGACGTGCAAATCGATCGTCAGATCCGGGTCTAGGGGC----------------------------------------------------------------------------------------------------------------------------------------------------------------------------------------------------------------------CTTTTCCTCGAATAAATAATCTTAGTTTTTGACTTCTTCCACCTTCTTTAATGTTATTATTTATTTCTTCTATAGCTGAAATAGGAGTTGGAGCAGGATGAACTATTTACCCTCCTTTAGCTTCTGTTGTAGGTCATAATGGAAGATCTGTAGATTTTGCTATTTTTTCTTTACATTTGGCAGGAGCGTCTTCTATTATGGGAGCTATTAATTTTATTTCTACAGTTATTAATATACGGTCTATAGATATAACTATAGATAAAATTACATTATTTGTTTGATCTGTTATTATTACAGCTGTTTTATTATTATTATCTTTACCTGTATTAGCAGGTGCTATTACTATATTGCTTACTGATCGAAATTTTAATACTTCTTTTTTTGATCCTGCAGGAGGTGGTGATCCTATTTTATTTCAGCATTTATTTTGATTTTTTGGACATCCTGAGGTCTATATCTTAATTCTTCCTGGTTTTGGGATTGTTTCACATATTATTAGTGCTTCTGTTGGAAAGCGTGAACCTTTTGGTTCATTAGGTATAATTTATGCTATAGTTGGAATTGGTGGAATAGGTTTTGTAGTGTGAGCTCACCATATATTTTCAGTGGGAATAGATGTTGATACTCGGGCTTATTTTACAGCTGCTACTATAATTATTGCGGTCCCTACTGGAATTAAAGTATTTAGATGAATAGCTACTCTTTATGGTTCTTATTTTAAGATAGATACCCCTTTGTTATGAAGTATTGGATTTGTATTTTTATTTACTTTAGGTGGAATTACAGGAGTTGTATTATCTAATTCTTCTTTAGATATTGTATTACATGATACTTATTATGTAGTAGCTCATTTTCATTATGTATTGAGAATAGGAGCTGTATTTGCTATTTTATCTGGAATTACTTATTGATTTCCTTTATTTTTTGGTGTTGTTCTTTGTGAAWAAAAANCTAAATTACAATTTTTGGTAATGTTTTTAGGTGTAAAT

>Bathippus directus JXZ205

TTTATAGTGAGATCTGCTCAATGA----TTA-TTAAATAGCCGCAATT-TGTGCTAAGGTAGCATAATCAT-TTGTCTTTTAAATGAAGACCAGAACAAAAGATTTAACATTTTAAATTATTTATTAAAATAACTAATTAAATTTATCCGAGATGTAAAAAGACATCTCTAAAAAAGAAAGACGATAAGACCCTATCGAACTTAACTTAAGTTTAACTGGGGTAGTAAATTAATTATTATTTTAATAAAAATTATTATTCAAATATCCATAT-ATTTTGATAATCCAGTTACCGTAGGGATAACAGCGCAATTAACTTTTAAAGTTCTTATTTAAAATATAGATTACGACCTCGATGTTGAATTAATATCC---TATTTT----AAGCAAATTTAAAAA-AAGAAAGTCTGTTCGACTTTTAATAAATTACATGATTTGAGTTCAGACCGGKGTAAGCCAGGTCGGTTTCAATCTTCTAAAAACTTTTTTACAGTACGAAAGGGCCTAAAAATATAATTTTATTTTATA-ATTGGCAGATTAATGCATTAGAATTAGAATCTAATTAAACTTACATTTATTTTTTATTAAATTATATTCTAATTTTAATTAGAATCTTAATTAGAGTAGCTTTCTATACTATTTTAGAACGAAAAGTATTAGGATATATTCAACTACGAAAAGGCCCTAATAAAGTAGGAATAATAGGAATTTTACAACC------------------------------------------------------------------------------------------------------------------------------------------------------------------------------------------------------------------------------------------------------------------------------------------------------------------------------------------------CCGGCGGCAAGTCCCCCTGACCGGGGCCTGTCCGTAGCGGGTGCGAGGCCCATAGCCGCCGGTCCCGT-CTCGAATGCGATCTCCTGGGAGTCGGGTTGCTTGGGAGTGCAGCCCTAATCGGGTGGTAAACTCCACCTAAGGCTAAATACCGCCGTGAGACCGATAGCAAACAAGTACCGTGAGGGAAAGTTGCAAAGAACTTTGAAGAGAGAGTTCAAGAGTACGTGAAACTGCTCAGAGGTAAACGGGTGGGCCCTCGAAATCCTGTGGCGAGAGGATTCAGTCTTGAGCGGAGGACTTC---CCGGGGGAAGAAACCG-AGCAGGGCGTCCGCGTCA-CGCGGGCCGTCCTCCGGGT-CG---ACCTCACCCGCCAAAGACGCATTTGACTCTCGCCCCGAGGACGCTGCAGCCGGTCTGGAAGTTGCCAGCGCGACGGCCTGAGGGCCGGGAAACTGGCAGGTGGCCGGTGGCTC-GCCGCG-TGCGGGTCGTCGGTTGT--TAGCCGTCCCGGCAGTGGCTCGTCTCCAGACCGAGGTGT-CGCGAGTCCCTTCG--GGGACTCCTCGTCTCCACCCC----AGAGTC-GCGGGACAGGGCACGGACCGGG-TCTT-CGCCGTCGA----CCTCCGCACTGGCACGGAGTGGGCGGAAGACACGGAGACCCAT--ACCGCCGTGACGGA--TCCGCGGGGGACCGGAGGCTGGCAGCGAGTAGGTCGGTCACCCACCCGACCCGTCTTGAAACACGGACCAAGGAGTCTAACATGTGCGCGAGTCAGTGGGTCTTGAACAGGCCCACGGGCGCAATGAAAGCGAAAGG-CCGCC-ATGCGCG-G---CCCGAGTGGGGATCCCCGCTCTTAACCGGGCGGGGCGCACCCACGACCCGTCCTATCCGGCTCGCCGGTTGGGCGGAGTCTGAGCGTACACGTTGGGACCCGAAAGATGGTGAACTATGCCCGGACAGGACGAGGCCAGAGGAAACTCTGGTGGAGGTCCGCAGCGGTTCTGACGTGCAAATCGATCGTCAGATCCGGGTCTAGGGGC-----------------------------------------------------------------------------------------------------------------------------------------------------------------------------------------------------------------------------------------------------------TCCCCCCTCTTTAATACTATTATTTATTTCTTCTATARCTGAAATAGGAGTAGGTGCGGGATRAACTGTTTATCCTCCTTTAGCTTCTGTTGTAGGACACAATGGAAGATCTGTGGATTTTGCTATTTTTTCTTTACATTTAGCGGGGGCGTCTTCTATTATGGGAGCTATTAATTTTATTTCTACGGTTATTAATATACGATCTATAGATATATCTATAGATAAGATTACATTATTTGTATGATCTGTTATCATTACGGCTGTTTTATTATTACTATCTTTACCTGTATTAGCAGGTGCTATTACTATATTACTTACAGATCGAAATTTTAATACTTCTTTTTTTGACCCTGCAGGGGGTGGTGACCCTATTTTATTTCAACATTTATTTTGATTTTTTGGACATCCTGAAGTTTATATTTTAATTCTTCCTGGTTTTGGTATTATTTCTCATATTATTAGTAGTTCTGTTGGAAAACGTGAACCTTTTGGTTCATTAGGAATAATTTATGCTATGGTTGGGATTGGTGGAATAGGTTTTGTAGTATGGGCTCATCATATATTTTCAGTAGGAATAGATGTTGATACTCGGGCTTATTTTACAGCTGCTACTATAATTATTGCTGTTCCCMCYGGAATTAGGGTATTTAGATGAATGGCTACTCTTTAKGGATCTTATTTTAAAATAGATGTTCCATTGTTAWGGAGTATTGGATTTGTATTTTTATTTACCTTAGGAGGAATTACAGGAGTTGTATTATCTAATTCTTCTTYGGATATTATTTTACATGATACTTATTATGTAGTASCTCATTTTCATTATGTATTAAGAATAGGGGCTGTATTTGCTATTT--------------------------------------------------------------------------------------------------

>Bathippus gahavisuka JXZ228

-----------------------------------------CGCAATT-CGTGCTAAGGTAGCATAATCAT-TTGTCTTTTAAATGAAGACTAGAACAAAAGATTTAATATTTTAATTTTTATATTTAAATATATAATCAAACCTTTACAAAATGTAAAAAGACATTTCTAAAAAAGAAAGACGACAAGACCCTATCGAACTTTACTTAAGTTTAACTGGGGCAGTTAATTAATAATTATTTTAATTTAAATTTAATTAATATTATCCAATT--AACTGATAATTAAGTTACCGTAGGGATAACAGCGCAATTAATTTTTAAAGCTCTTATTTAAAAAATAGATTACGACCTCGATGTTGAATTAATAACCTATCTTTTA----AAGCAAATTTTAGAATAAGGGAGTCTGTTCGACTCATAACAAATTACATGATTTGAGTTCAGACCGGTATAAGCCAGGTCGGTTTCAATCTTCTAAAAATCTTTTTACAGTACGAAAGGACCTAAAAATCTAATTTAATTAATTA-ATTGGCAGAAAAATGCATTAGAATTAGAATCTAATTAAACTAATATTTACTTCACAGTAAATTATATTCTAATTTTAATTAGAATTTTAATTAGTGTAGCTTTTTACACTATTTTAGAACGAAAAGTATTAAGATATATCCAATTACGAAAAGGTCCTAATAAAGTAGGAATATTAGGGATTTTACAACCTTTTAGAGATGCCTTAAAACTTTTTAATAAAAATTTAGTATCACCTGAAACAATAAATTTTATTTTTTCTTATTTAACCCCCGCTATATCTCTAATAATTAGATTATTATTAATTCCTATTATTATATATAATAATATATCATTATATGATAATAAACATAATATTTTATTCTTTTTTATTCTTTCCAGAATTTCTGTGTATTCAATTTTATTAATTGGATGATCA---------------------------------------------------------------------------------GTTTAGGAGTGAGCGTGTCGAGTCCGGAGCCGGCGGCAAGTCCCCCTGACCGGGGCCTGTCCGTAGCGGGTGCGAGGCCCATAGCCGCCGGTCCCGT-CTCGAATGCGATCTCCTGGGAGTCGGGTTGCTTGGGAGTGCAGCCCTAATCGGGTGGTAAACTCCACCTAAGGCTAAATACCGCCGTGAGACCGATAGCAAACAAGTACCGTGAGGGAAAGTTGCAAAGAACTTTGAAGAGAGAGTTCAAGAGTACGTGAAACTGCTCAGAGGTAAACGGGTGGGCCCTCGAAATCCTGTGGCGAGAGGATTCAGTCTTGAGCGGAGGACTTC---CCGGAGGAAGAAACCG-AGCAGGGCGTCCGCATCA-CGCGGGCCGTCCTCCGGGT-CG---ACCTCAACCGCCAAAGACGCATTTGACTCTCGCCCCGAGGACGCTGCAGCCGGTCTGGAAGTTGCCAGCGCGACGGCCTGAGGGCCGGGAAACTGGCAGGTGGCCGGTGGCTC-GCCGCG-TGCGGGTCGTCGGTTGT--TAGCCGTCCCGGCAGTGGCTCGTCTCCAGACCGAGGTGT-CGCGAGTCCCTTCG--GGGACTCCTCGTCTCCACCCC----AGAGTC-GCGGGACAGGGCACGGACCGGG-TCTT-CGCCGTCGA----CCTCCGCACTGGCACGGAGTGGGCGGAAGACACGGAGACCCAT--ACCGCCGTGACGGA--TCCGCGGGGGACCGGAGGCTGGCAGCGAGTAGGTCGGTCACCCACCCGACCCGTCTTGAAACACGGACCAAGGAGTCTAACATGTGCGCGAGTCAGTGGGTCTTGAACAGGCCCACGGGCGCAATGAAAGCGAAAGG-CCGCC-GAGCGCG-G---CCCGAGTGGGGATCCCCGCTCTTCGCCGGGCGGGGCGCACCCACGACCCGTCCTATCCGGCTCGCCGGTTGGGCGGAGTCTGAGCGTACACGTTGGGACCCGAAAGATGGTGAACTATGCCCGGACAGGACGAGGCCAGAGGAAACTCTGG------------------------------------------------------------------------------------------------------------------------------------------------------------------------------------------------------------------------------------------------------------------------------------------ATAAATAATTTAAGTTTTTGGTTATTACCACCGTCCTTAATATTGTTATTTATTTCTTCTATGGTTGAGATAGGTGTGGGATCTGGATGAACTGTTTATCCACCTTTAGCTTCTATTGTTGGTCATAATGATAGATCTGTGGATTTTGCTATTTTTTCTTTACATTTAGCGGGAGCATCATATATTATAGGGGCTATTAATTTTATTTCTACAGTTATTAATATACGATCTATAGATATATCTATAGATAAAGTTACTTTATTTGTGTGATCTGTAATTATTACAGCAATTTTATTATTATTGTCTTTACCTGTTTTAGCAGGTGCTATTACTATATTACTTACTGATCGAAATTTTAATACTTCTTTTTTTGACCCTGCAGGTGGAGGGGATCCTATTTTGTTTCAGCATTTATTTTGATTTTTTGGTCATCCYGAAGTTTATATTTTAATTCTTCCKGGTTTTGGAATTGTTTCTCATATTATTAGTGCTTCYGTAGGAAAACGTGAACCATTTGGTTCGTTAGGTATAATTTATGCTATAATTGGAATTGGAGGAATAGGATTTGTAGTATGAGCACATCATATATTTTCAGTAGGAATAGATGTAGATACTCGAGCTTATTTTACAGCTGCTACAATAATTATTGCAGTCCCAACTGGAATTAAAGTATTTAGTTGAATAGCTACTCTTTATGGTTCTTATTTTAAGATAGATACTCCTTTAATATGAAGTATTGGGTTTGTATTCTTATTTACTTTAGGAGGAATTACKGGAGTAGTATTATCTAATTCTTCTTTAGATATTGTTTTACATGATACTTATTAKGKGGTAGCMCATTTTCWTTATGTATTAAGAATAGGGGCGGTATTTGCTATTTTAGCGGGAATTACYYATTGATTTCCTTTATTTTTTGGTATAGTTCTTNKNGAAAAAAAAACTAAATTACAATTTTTAGTAAT----------------

>Bathippus korei JXZ284

TTAGTAGTAAAGTCTGCTCCATGA----ATACTTAAATAGCCGCAATT-CGTGCTAAGGTAGCATAATCAT-TTGTCTTTTAAATGAGGACTAGAACAAAAGATTTAACATTTTAAATTATTTATTTAAATTAATTATTTAAATTTTCTTAAGCGTAAAAAGACACTTATTAAAAAGAAAGACGACAAGACCCTATCGAACTTAACTCAAGTTTAACTGGGGTAGTTAATTAATTATTATTTTAATATACTATAACTACTAATTATCTAATT--AATTAATAATCCAGTTACCGTAGGGATAACAGCGCAATTAAATTTTAAAGTACTTATTTAAAAAATAGATTACGACCTCGATGTTGAATTAATAACC---TATTTG----AAGCAAATTTCAAAA-AAGGAAGTCTGTTCGACTTTTAAAAAATTACATGATTTGAGTTCAGACCGGTATAAGCCAGGTCGGTTTCAATCTTCTAAAAACTCTTTTACAGTACGAAAGGACCTAAAAATATAATTTAATTATTTATATTGGCAGAAAAATGCATTAGAATTAGAATCTAATAAAACTTTTATCCTATTTTCTTTAAATTATATTCTAATTTTAATTAGAATCTTAATTAGCGTAGCTTTTTACACTATTTTAGAACGAAAAATATTAAGCTATATTCAAACCCGGAAAGGGCCTAATAAAGTAGGAATAATAGGGATTTTACAACC---------------------------------------------------------------------------------------------------------------------------------------------------------------------------------------------------------------------------------------CCCTGAGTAACGGCGAGCGAAAAGGGATGAGCCCAGCGCCGAATCCCCGGCC--TTCCGGTCGCGGGAAATGTGGCGTTTAGGAGTGAGCGTGTCGAGTCCGGAGCCGGCGGCAAGTCCCCCTGACCGGGGCCTGTCCGTAGCGGGTGCGAGGCCCATAGCCGCCGGTCCCGT-CTCGAATGCGATCTCCTGGGAGTCGGGTTGCTTGGGAGTGCAGCCCTAATCGGGTGGTAAACTCCACCTAAGGCTAAATACCGCCGTGAGACCGATAGCAAACAAGTACCGTGAGGGAAAGTTGCAAAGAACTTTGAAGAGAGAGTTCAAGAGTACGTGAAACTGCTCAGAGGTAAACGGGTGGGCCCTCGAAATCCTGTGGCGAGAGGATTCAGTCTTGAGCGGAGGACTTC---TCGGAGGAAGAAACCG-AGCAGGGCGTCCGCGACA-CGCGGGCCGTCCTCCGAGT-CG---ACCTCAGCCGCCAAAGACGCATTTGACTCTCGCCCCGAGGACGCTGCAGCCGGTCTGGAAGTTGCCAGCGCGACGGCCTGAGGGCCGGGAAACGGGCAGGTGGCCGGTGGCTC-GCCGCG-TGCGGGTCGTCGGTTGT--TAGCCCTCCCGGCAGTGGCTCGTCTCCAGACCGAGGTGT-CGCGAGTCCCTTCG--GGGACTCCTCGTCTCCACCCC----AGAGTC-GCGGGACAGGGCACGGACCGGG-TCTT-CGCCGTCGA----CCTCCGCACTGGCACGGAGTGGGCGGAAGACACGGAGACCCAT--TCCACCGTGACGGA--CCCGCGGGGGACCGGAGGCTGGCAGCGAGTAGGTCGGTCACCCACCCGACCCGTCTTGAAACACGGACCAAGGAGTCTAACATGTGCGCGAGTCAGTGGGTCTTGAACAGGCCCACGGGCGCAATGAAAGCGAAAGG-CCGCC-GAGCGCG-G---CCCGAGTGGGGATCCTCGCTCTTCGCGGGGCGGGGCGCACCCACGACCCGTCCTATCCGGCTCGCCGGTTGGGCGGAGTCTGAGCGTACACGTTGGGACCCGAAAGATGGTGAACTATGCCCGGACAGGACGAGGCCAGAGGAAACTCTGG----------------------------------------------------------------------------------------------------------------------------------------------------------------------------------------------------------------------------------------------------------------------------G--CTTTTCCTCGTATAAATAATCTTAGTTTTTGATTATTACCTCCTTCTTTANTATTATTATTTATTTCTTCTATAGCTGAAATAGGAGTTGGTTCAGGATGAACTGTCTATCCTCCTTTAGCTTCTATTGTTGGTCATAATGGTAGTTCTGTGGATTTTGCTATTTTTTCTTTACATTTGGCTGGAGCGTCTTCTATTATAGGTGCTATTAATTTTATTTCTACGGTTATTAACATACGATCTGTTGATATATCTATAGATAAAATTACATTATTTGTATGGTCTGTAATTATTACAGCTGTTTTATTATTATTGTCTTTACCTGTATTGGCAGGTGCTATTACTATATTACTTACGGATCGTAATTTTAATACTTCTTTTTTTGACCCTGCAGGAGGAGGTGATCCTATTTTATTTCAACATTTATTTTGATTTTTTGGACACCCTGAAGTTTATATTTTAATTCTTCCTGGGTTTGGAATTGTGTCTCATGTAATTAGTGCTTCTGTAGGAAAGCGTGAACCATTTGGATCATTAGGTATAATTTATGCAATAGTTGGAATTGGAGGAATAGGATTTGTAGTGTGAGCTCATCATATATTTTCGGTAGGTATGGATGTAGATACTCGGGCTTATTTTACAGCTGCTACTATAATTATTGCAGTTCCTACTGGAATTAGGGTATTTAGATGAATAGCTACTCTTTATGGTTCTTATTTTAAAATAGATACTCCTTTATTGTGAAGAATTGGTTTCGTATTTCTTTTTACTTTAGGAGGAATTACGGGAGTAGTTTTGTCTAATTCTTCTTTAGATATTATTTTACATGACACTTATTATGTGGTGGCTCATTTTCATTACGTATTAAGAATAGGAGCGGTATTTGCTATTTTAGCAGGGATTACTTATTGATTTCCTTTATTTTTTGGAGTGGTTCTTTGTGAAANNAAANCTAAGTTACAATTTTTGGTTATATTTTTAGGTGTAAAT

>Bathippus madang JXZ143

TTAATAATAAAATCTGCTCCATGA----ATA-TTAAATAGCCGCAATT-TGTGCTAAGGTAGCATAATCAT-TTGTCTTTTAAATGAAGACTAGAACAAAAGATTTAACATTTCAACTTATTTATTTATAAAAACAATCAAATTTTTCTAAAATGTAAAAAGACATTTATAAGTCAGAAAGACGACAAGACCCTATCGAACTTTACTTTAGTTTTACTGGGGTAGTAAATTAATTATTATTTTAATATAATTATTTATATAAATATCCATTT--ATATGATAATCAAGTTACCGTAGGGATAACAGCGCAATTAAATTTTAAAGACCTTATTTAAAAAATAGATTACGACCTCGATGTTGAATTAATAACC---TTTTTA----AAGCAAATTTTAGAA-AAGGAAGTCTGTTCGACTTTTAATAAATTACATGATTTGAGTTCAGACCGGTATAAGCCAGGTCGGCTTCAATCTTCTAAAAACTCTTTTACAGTACGAAAGGACCCAAAAATATAATTTAATTAATTA-ATTGGCAGATTAATGCATTAGAATTAGAATCTAATAAAACTTCTATTCCATTCTTTCTTAATTATATTTTAATTCTAATTAGAATCCTAATCAGTGTAGCATTCTACACTATTCTAGAACGAAAAATTCTAAGATATATTCAAGTCCGAAAAGGACCTAATAAAGTAGGGCTTATAGGAATTCT---------------------------------------------------------------------------------------------------------------------------------------------------------------------------------------------------------------------------------------------------AGTACGGCGAGCGAAAAGGGATGAGCCCAGCGCCGAATCCCCGGCC-TTACCGGTCGAGGGAAATGTGGCGTTTAGGAGTGAGCGTGTCGAGTCCGGAGCCGGCGGCAAGTCCCCCTGACCGGGGCCTGTCCGTAGCGGGTGCGAGGCCCATAGCCGCCGGTCCCGT-CTCGAATGCGATCTCCTGGGAGTCGGGTTGCTTGGGAGTGCAGCCCTAATCGGGTGGTAAACTCCACCTAAGGCTAAATACCGCCGTGAGACCGATAGCAAACAAGTACCGTGAGGGAAAGTTGCAAAGAACTTTGAAGAGAGAGTTCAAGAGTACGTGAAACTGCTCARAGGTAAACGGGTGGGCCCTCGAAATCCTGTGGCGAGAGGATTCAGTCTTGAGCGGAGGACTTC---CCGGAGGAAAAAACCATAGCAGGGCGTCCGCGTCG-CGCGGGCCGTCCTCTGGGT-CG---ACCTCTGCCGCCAAAGACGCATTTGACTCTCGCCCCGAGGACGCTGCAGCCGGTCTGGAAGTTGCCAGCGCGACGGCCTGAGGGCCGGGAACCGGGCAGGTGGCCGGTGGCTC-GCCGCG-TGCGGGTCGTCGGTTGT--TAGCCCTCCCGGCAGTGGCTCGTCTCCAGACCGAGGTGT-CGCGAGTCCCTTCG--GGGACTCCTCGTCTCCACCCC----AGAGTC-GCGGGACAGGGCACGGACCGGG-TCTT-CGCCGTCAA----CCTCCGCACTGGCACGGAGTGGGCGGAAGACACGGAGACCCAA--TCCGCCGTGACGGA--CCCGCGGGGGACCGGAGGCTGGCAGCGAGTAGGTCGGTCACCCACCCGGCCCGTCTTGAAACACGGACCAAGGAGTCTAACATGTGCGCGAGTCAGTGGGTCTTGAACAGGCCCACGGGCGCAATGAAAGCGAAAGG-CCGCC-GAGCGCG-G---CCCGAGTGGGGATCCTCGCCCTTCGCGGGGCGGGGCGCACCCACGACCCGTCCTATCCGGCTCGCCGGTTGGGCGGAGTCTGAGCGTACACGTTGGGACCCGAAAGATGGTGAACTATGCCCGGACAGGACGAGGCCAGAGGAAACTCTGGTGGAGGTCCGCAGCGGTTCTGACGTGCAAATCGATCGTCAGATCCGGGTCTA--------------------------------------------------------------------------------------------------------------------------------------------------------------------------------------------------------------------------------------------------------------------------------------------------------------------------------------------------------------------------------------------------------------------------------------------------------------------------------------------------------------------------------------------------------------------------------------------------------------------------------------------------------------------------------------------------------------------------------------------------------------------------------------------------------------------------------------------------------------------------------------------------------------------------------------------------------------------------------------------------------------------------------------------------------------------------------------------------------------------------------------------------------------------------------------------------------------------------------------------------------

>Bathippus macrognathus JXZ372

--------------------ATGA----TTAATTAAATAGCCGCAATT-TGTGCTAAGGTAGCATAATAAT-TTGTCTTTTAAATAAAGACCAGAACAAAAGATTTAACATTTTAATCTATTTATTTAAAATAAAAATTCAAATTTTCTTAAATGTAAAAAGACATTTCTATAAAAGAAAGACGACAAGACCCTATTGAACTTAACTTTAGTTAAACTGGGGCAGTTAATTAAATATTACTTTAATTCATCTTTTTTCATAATAATCCAAAATTATTTGATAATTCAGTTACCATAGGGATAACAGCGCAATTAAATTTTAAAGCTCTTATTTAAAATATAGATTACGACCTCGATGTTGAATTAATAACC---TATATA----AAGCAAATTTCAAAA-AAGGAAGTCTGTTCGACTTTTAATTAATTACATGATTTGAGTTCAGACCGGTGTAAGCCAGGTCGGTTTCAATCTTCTATAAACTTTTTTACAGTACGAAAGGACCTAAAAATATAATTTAATTAATTA-TTTGGCAGATCAATGCATTAGAATTAGAATCTAATAAAACTTATAT---ATTTATAATTAATTATCTATTAGTATTAATTAGAATTCTAATTAGAGTAGCTTTTTACACAATTTTAGAACGAAAAATTTTAGGATATATCCAAATTCGAAAAGGTCCCAATAAAGTAGGAATAATAGGAATTTTACAACCTTTTAGAGATGCATTAAAACTTTTTAACAAAAATATTATCTCCTYTGAAAATATAAACTTTGTATTATYTTATACAACTCCAGCTATATCCTTAATAATTAGAATATTACTAATTCCAATTATTTTATATAACCATATATCATTATATGACAACAAACATAACATTCTCTTATTTTTTAT----------------------------------------------------CCTGAGTAACGGCGAGCGAAAAGGGATGAGCCCAGCGCCGAATCCCCGGCC-CTTCGGGTCGCGGGAAATGTGGCGTTTAGGAGTGAGCGTGTCGAGTCCGGAGCCGGCGGCAAGTCCCCCTGACCGGGGCCTGTCCGTAGCGGGTGCGAGGCCCATAGCCGCCGGTCCCCT-CTCGAATGCGATCTCCTGGGAGTCGGGTTGCTTGGGAGTGCAGCCCTAATCGGGTGGTAAACTCCACCTAAGGCTAAATACCGCCGTGAGACCGATAGCAAACAAGTACCGTGAGGGAAAGTTGCAAAGAACTTTGAAGAGAGAGTTCAAGAGTACGTGAAACTGCTCAGAGGTAAACGGGTGGGCCCTCGAAATCCTGTGGCGAGAGGATTCAGTCTGGAGCGGAGGACTTCT--CCGGAGGAAGAAACCG-AGCAGGGCGTCCGCACCA-CGCGGGCCGTCCTCCGGGT-CG---CCCTCTGCCGCGAAAGACGCATTTGACTCTCGTCCCGAGGACGCTGCAGCCGGTCTGGAAGTTGCCAGCGCGACGGCCTGAAGGCCGGGAGACAGGCAGGTGGCCGGTGGCTC-GCCACGTTGCGGGTCGTCGGTTGT--TAGCCATCCCGGCAGTGGCTCGTCTCTAGACCGTGGTGT-CGCGAGTCCCTTCAC-GGGACTCCTCGTCTCCTCCCCC--AGAGATC-GCGGGACAGGGCACGGACCGGG-TCTT-CGCCGTCGA----CCTCCGCACTGGCACGGAGTGGGCGGAAGATTCGGAGACCCAA--TCCGCCGTGACGGA--CCCGCGGGGGACTGGAGGCTCGCAGCGAGTAGGTCGGTCACCCACCCGACCCGTCTTGAAACACGGACCAAGGAGTCTAACATGTGCGCGAGTCAGTGGGTCTTGAACAGGCCCACGGGCGCAATGAAAGCGAAAGG-CCGCC-GAGCGCGTG---CCCGAGTGGGGATCCCGGCTCCCAACAGGGCCGGGCGCACCCACGACCCGTCCCGTCCGGCTCGCCGGTGGGGCGGAGTCTGAGCGTACACGTTGGGACCCGAAAGATGGTGAACTATGCCCGGACAGGACGAGGCCAGAGGAAACTCTGGTGGAGGTCCGCAGCGGTTCTGACGTGCAAATCGATCGTCAGATCCGGGTCTAGGGG---------------------------------------------------------------------------------------------------------------------------------------------------------------------------------------------------------------------------------------TAATTTAAGCTTTTGATTGTTACCTCCATCTTTAATATTGTTGTTTGTTTCTTCTATATCTGAAATGGGGGTAGGTTCTGGATGAACGGTTTATCCCCCTTTAGCTTCTATTATTGGTCATAATGGAAGCTCAGTGGATTTTGCTATTTTTTCTTTACATTTGGCTGGAGCATCTTCTATTATAGGAGCTATTAATTTTATTTCTACTGTTATTAATATACGATCTGTTGATATATCGATGGATAAAATTACTTTGTTTGTGTGGTCCGTAATTATTACAGCTGTTTTATTGTTGTTATCTTTACCAGTTTTAGCTGGAGCTATTACTATATTACTTACAGATCGAAATTTTAATACTTCTTTTTTTGATCCTGCAGGAGGGGGGGATCCAGTGTTATTTCAACATTTATTTTGATTTTTTGGACATCCTGAGGTTTATATTTTAATTCTTCCCGGTTTTGGAATTGTTTCGCATGTTATTAGTGCTTCTGTTGGAAAGCGTGAACCGTTTGGTTCATTAGGTATAATTTACSCAATAGTTGGAATTGGTGGAATAGGATTTGTAGTATGAGCTCATCMCATATTTTCGGTAGGTATAGACGTGGATACTCGGGCTTATTTTACTGCGGCTACTATAATTATTGCGGTTCCAACTGGAATTAAAGTATTTAGATGGATAGCAACTCTTTATGGATCTTATTTCAAAATTGATACTTCATTGATATGAAGTATCGGTTTTGTATTTTTATTTACTTTGGGTGGTATTACTGGGGTTGTATTGTCCAATTYTTCATTAGATATTGTYTTACATGATACTTATTATGTGGTGGCTCATTTTCATTATGTATTGAGAATAGGTGCGGTATTTGCTATTTTAGCCGGTATTACTTATTGATTTCCTTTATTTTTTGGTGTGGCTCTTTGTGAAAAAAAAACTAAATTACAATTTTTAGTAATATTTTTAGGAGTGAA-

>Canama extranea JXZ258

-----------------------------------------------------------------------------TTTTAAATAAAGACTAGAACAAAAGATTTAACATTTTAATTTTTTTATTTGAATTATTTATCAAAATTATTTTATATGTAAAAATACATATATGTAAAAGAAAGACGACAAGACCCTATTGAACTTTACTTAAGTTTAACTGGGGCAGTTAGTTAATTATTATTTTAACTTATCACTCTCAAAAAAAAACCATAA--CTTTGATAAATCAGTTACCATAGGGATAACAGCGCAATTAAATTTTAAAGCTCTCATTTAAAAAATAGATTACGACCTCGATGTTGAATTAATTTTC---TATTTA----AAGCAAATTTTAAAA-AAGAAAGTCTGTTCGACTTATAACAAATTACATGATTTGAGTTCAGACCGGCGTAAGCCAGGTCGGTTTCAATCTTCTAAAAACTTTTTAACAGTACGAAAGGACCTTAAAATATAATTAAATTAATTA-ATTGGCAGATTAATGCATTAGGTTTAGAATCTAATAAAACTTAAATATTATTTATATTTAATTATATCTTCGTATGTATTAGAATTCTTATTAGAGTAGCCTTTTATACAATTTTAGAACGAAAAATCCTAGGATATATTCAAATTCGAAAAGGACCAAATAAAGTTGGTACAATAGGAATTTTACAAC--------------------------------------------------------------------------------------------------------------------------------------------------------------------------------------------------------------------------------------------------------------------------------------------------------GGGAAATGTGGCGTTTAGGAGTGAGCGTGTCGGGTTCGGATGCGGCGGCAAGTCCCCCTGACCGGGGCCTGTCCGTAGCGGGTGCGAGGCCCATAGCCGCCGCTCCCGT-CCCGAATGCGATCTCCTGGGAGTCGGGTTGCTTGGGAGTGCAGCCCTAATCGGGTGGTAAACTCCACCTAAGGCTAAATACTGCCGTGAGACCGATAGCAAACAAGTACCGTGAGGGAAAGTTGCAAAGAACTTTGAAGAGAGAGTTCAAGAGTACGTGAAACTGCTCAGAGGTAAACGGGTGGGCCCTCGAAATCCTGTGGCGAGAGGATTCAGTCTGGATCGGAGGATTCCG--GTGGCGGAAGTAA-CG-TGCAGGGAGTCCGCACCATTGCGGGCCGTCCGCCTCG--GA---ACCTCATCCGTGACAGACGCATTTGACTCTCGTCCGATGGACGCTGCAGCCGGTTTGGAAGTTGCCAGCGCGACGGCCTGAAGGCCGGGAGACAGGCAGGTGGCCGGTGGCTC-GCCTCG-TGCGGGTCGTCGGTTGT--TAGCCTTCCCGGTAGTGGCTCGTCTCCAGACCGAGGTGT-CGCGAGTCCCTTAAC-GGGACTCCTCGTCTCCTCCCC----AGAGTC-GTGGAACAGGGCGCGGCCCGGG-TCTT-CGCCGTCGA----CCTCCGCACTGGCACGGAGTGGGCGGGAGAGTCGGAGACCCAA--ACCGCCGCGACGGA--TCCGCGGGGGACTGGAGGCTCGCAGCGAGTAGGTCGGTCACCCACCCGACCCGTCTTGAAACACGGACCAAGGAGTCTAACATGTGCGCGAGTCAGTGGGTCTTGAACAGGCCCACGGGCGCAATGAAAGCGAAGGG-CTTCC-GAGCGTT-G---CCCGAGTGGGGATCCCCGCACCTCG--GTGTGGGGCGCACCCACGACCCGTCCATTCCGGCTCGCCGGTCGGGCGGAGTTTGAGCGTACACGTTGGGACCCGAAAGATGGTGAACTATGCCCGGACAGGACGAGGCCAGAGGAAACTCTGGTGGAGGTCCGCAGCGGTTCTGACGTGCAAATCGATCGTCAGATCCGGGTCTAGGGGC----------------------------------------------------------------------------------------------------------------------------------------------------------------------------------------------------------------------CTTTTCCTCGTATAAATAATCTTAGTTTTTGATTGTTACCGCCTTCTTTAATATTACTTTTTGTTTCTTCTATAGTGGAGGTAGGAGTGGGTTCAGGTTGAACCGTATATCCCCCTTTGGCCTCTATTGTAGGTCATAATGGAAGATCTGTGGATTTTGCTATTTTTTCTTTACATTTAGCTGGTGCTTCTTCTATTATGGGAGCTATTAATTTTATTTCTACTGTAATTAATATACGATCTATTGATATATCTTTGGATCGTATAACTTTATTTGTGTGGTCTGTGGTAATTACGGCTGTATTATTATTATTATCTTTACCTGTTTTAGCAGGGGCTATTACAATATTGTTGACAGATCGAAATTTTAACACTTCTTTTTTTGATCCTGCTGGTGGAGGTGATCCTATTTTATTTCAACATTTATTTTGATTTTTTGGACATCCTGAGGTTTATATTTTAATTTTACCTGGATTTGGAATTGTTTCTCATGTGATTAGTGCTTCTGTAGGAAAGCGTGAACCATTTGGTTCATTAGGTATAATCTATGCTATAGTTGGAATTGGAGGAATAGGTTTTGTAGTGTGAGCTCATCATATATTTTCTGTAGGAATAGACGTTGATACGCGGGCATATTTTACGGCTGCTACAATAATTATTGCTGTTCCTACAGGAATTAAAGTATTTAGTTGAATAGCTACTCTTTATGGTTCTTATTTTAAAATTGATACTCCATTAATGTGAAGTATTGGGTTTGTATTTTTATTTACATTAGGTGGAATTACGGGAGTTGTTTTGTCTAATTCTTCTTTAGATATTGTTTTACATGATACTTACTATGTAGTTGCACATTTTCATTATGTATTGAGAATAGGAGCTGTTTTTGCTATTTTGGCTGGAATTACTTATTGATTTCCTTTATTTTTTGGAGTGGTTTTAAGTGAAAAGANGACTAGATTACAATTTATGGTAATATTTTTAGGAGTAAAT

>Canama fimoi JXZ265

------------------------------------------------------------------ATAAT-TTGTCTTTTAAATGAAGACTAGTACAAAAGATTTCACATTTTAAGTTTTTTATTTAGGATAAAATTCCAAATTCTCCTAAGTGTAAAAAGACACTTCTAAAAAAGAAAGACGACAAGACCCTATCGAACTTCACTTTAGTTTTACTGGGGAAGTAAATTATTTATTATTTTAATATATAATTCTATACAAATATCCACCT--AACTGATAAATCAGTTACCATAGGGATAACAGCGCAATTAAATTTTAAAGACCTTATTTAAAAAATAGATTACGACCTCGATGTTGAATTAATATCC---TTTTTA----AAGCAAATTTTAGAA-AAGAAAGTCTGTTCGACTTTTAATAAATTACATGATTTGAGTTCAGACCGGTGTAAGCCAGGTCGGTTTCAATCTTCTAAAAACTTTTTAACAGTACGAAAGGACCTAAAAATATAATATAATTATTAT-ATTGGCAGAAAAATGCATTAGAATTAGAATCTAATAAAACTAACATTACATTTATAATTAATTACATTATTGTATGTATTAGAATTTTAATTAGAGTAGCTTTCTACACCATTTTAGAACGAAAGATCTTAGGATATATCCAAATCCGTAAAGGCCCTAACAAAGTTGGTATAACAGGGTTATTACAA-----------------------------------------------------------------------------------------------------------------------------------------------------------------------------------------------------------------------------------------------------------------------------------------------------------------------------------GTGTCGGGTTCGGAGGCGGCGGCAAGTCCCCCTGACCGGGGCCTGTCCGTAGCGGGTGCGAGGCCCATAGCCGCCGCTCCCGT-CCCGAATGCGATCTCCTGGGAGTCGGGTTGCTTGGGAGTGCAGCCCTAATCGGGTGGTAAACTCCACCTAAGGCTAAATACCGCCGTGAGACCGATAGCAAACAAGTACCGTGAGGGAAAGTTGCAAAGAACTTTGAAGAGAGAGTTCAAGAGTACGTGAAACTGCTCAGAGGTAAACGGGTGGGCCCTCGAAATCCTGTGGCGAGAGGATTCAGTCTGGATCGGAGGATTCCG--CGGGCGGAAGTAACCG-AGCAGGGCGTCCGCGTCACCGCGGACCGTCCGCCTCG--GA---ACCTCAATCGTGACAGACGCATTTGACTCTCGTCCGATGGACGCTGCAGCCGGTTTGGAAGTTGCCAGCGCGACGGCCTGAAGGCCGGGAGACAGGCAGGTGGCCGGTGGCTC-GCCTCG-TGCGGGTCGTCGGTTGT--TAGCCTTCCCGGTAGTGGCTCGTCTCCAGACCGAGGTGT-CGCGAGTCCCTTAAC-GGGACTCCTCGTCTCCGCCCC----AGAGTC-GTGGAACAGGGCACGGCCCGGG-TCTT-CGCCGTCAA----CCTCCGCACTGGCACGGAGTGGGCGGAAGAGACGGAGACCCTA--ACCGCCGTGACGGA--TCCGCGGGGGATCGGAGGCTCGCAGCGAGTAGGTCGGTCACCCACCCGACCCGTCTTGAAACACGGACCAAGGAGTCTAACATGTGCGCGAGTCAGTGGGTCTTGAATAGGCCCACGGGCGCAATGAAAGCGAAGGG-CTTCC-GAGCGTT-G---CCCGAGTGGGGATCCCTGCGCCTTGT-GCGCGGGGCGCACCCACGACCCGTCCATTCCGGCTCGCCGGTCGGGCGGAGTTTGAGCGTACACGTTGGGACCCGAAAGATGGTGAACTATGCCCGGACAGGACGAGGCCAGAGGAAACTCTGGTGGAGGTCCGCAGCGGTTCTGACGTGCAAATCGATCGTCAGATCCGGGTCTAGGGGCG--------------------------------------------------------------------------------------------------------------------------------------------------------------------------------------------------------------------------------------AATTTGAGTTTTTGATTATTACCCCCTTCTTTAATACTATTATTTATTTCTTCTATAGCAGAAATAGGTGTAGGATCTGGATGGACTGTGTATCCTCCTTTGGCATCTATTATTGGACATAATAATAGATCGGTAGATTTTGCTATTTTTTCCTTACATTTAGCTGGGGCTTCATCTATTATAGGGGCTATTAATTTTATTTCTACAGTTATTAATATACGATCTGTAGATATATCTATGGATAAAATTACTTTATTTGTATGATCTGTTGTAATTACTGCTGTATTATTACTTTTATCGTTGCCTGTTTTAGCAGGGGCTATTACTATACTGTTAACGGACCGAAATTTTAATACTTCTTTTTTTGATCCAGCAGGTGGGGGAGATCCTATTTTATTTCAACATTTATTTTGGTTTTTTGGACATCCTGAGGTATACATTTTGATTTTGCCTGGGTTTGGAATTGTTTCTCATGTGATTAGTGCTTCGGTAGGAAAGCGAGAACCATTTGGTTCTTTGGGTATAATCTATGCTATAGTAGGAATTGGGGGGATAGGATTTGTAGTATGGGCGCATCATATATTTTCGGTTGGAATAGATGTTGATACTCGGGCCTATTTTACTGCAGCTACTATGATTATTGCTGTTCCAACTGGAATTAAGGTATTTAGATGAATAGCTACTTTGTATGGATCATATTTCAAGATTGATACTCCGTTGATATGAAGTATTGGATTTGTATTTTTATTTACTTTGGGAGGAATTACTGGAGTGGTATTATCTAATTCTTCACTAGATATTGTTTTACATGATACTTATTATGTAGTGGCTCATTTTCATTATGTATTAAGAATAGGTGCCGTGTTTGCTATTTTAGCTGGGATTACTTATTGATTTCCTTTGTTTTTTGGTGTAGTATTAAATGAAAGGAAAACTAAGTTGCAATTCTTGGTAATATTTGTAGGAGT----

>Canama forceps JXZ210

TTAATAGTAAAATCTGCTCAATGAATTTTTAAATAAATAGCCGCAACT-CGTGCTAAGGTAGCATAATCAT-TTGTCTTTTAAATGAAGACTGGAACAAAGGATTTAACATTTCAATTTATTTATTTAGCTTAACAATTTAAATTATCTTAAATGTAAAAAGACATTTATAAAAAAGAAAGACGACAAGACCCTATTGAACTTAACTTTAGTTAAACTGGGGTAGTTAATTAATTATTATTTTAATTAAATTATCTTAAAAAAAATCCACTT--ACTTGATAATCAAGTTACCATAGGGATAACAGCGCAATTAAATTTTAAAGTCCTCATTTAAAAAGTAGATTACGACCTCGATGTTGAATTAATAACCTA-TATTTA----AAGTAAATTTTAAAA-AAGGAAGTCTGTTCGACTTTTAATAAATTACATGATTTGAGTTCAGACCGGTGTAAGCCAGGTCGGTTTCTATCTTCTATATAGTTTTCTACAGTACGAAAGGACCTAAAAATATAATTTAATTAATTA-ATTGGCAGATAAGTGCATTAGAATTAGAATCTAAATAATCTATTATATTATTCTTAATTAATTATATCTTAGTACTTATTAGAATTTTAATTAGAGTCGCTTTTTACACTATTTTAGAACGGAAAGTTCTAGGCTATATTCAAATTCGAAAAGGACCTAATAAAGTAGGAATACTTGGAATTCTCCAACCTTTCAGAGATGCTTTAAAACTTTTTAATAAAAATCTTTTGCCTTCAGAACATATAAATATTATATTGTGCTATATAATACCTGCTTTTTCTCTTTTAATTAGAATATTATTAATTCCAATTATTTTATATAATATATCAACATTATATGACAATAGGCATAATATCCTTTTATTCTTTGTTTTATCTAGTTTATCAGTTTATTCAATTTTATT----------------------------------------------------------------------------------------------------------------------------------------------------------------------------------------------------------------TTGGAGTCGGGTTGCTTGGGAGTGCAGCCCTAATCGGGTGGTAAACTCCACCTAAGGCTAAATACCGCCGTGAGACCGATAGCAAACAAGTACCGTGAGGGAAAGTTGCAAAGAACTTTGAAGAGAGAGTTCAAGAGTACGTGAAACTGCTCAGAGGTAAACGGGTGGGCCCTCGAAATCCTGTGGCGAGAGGATTCAGTCTGGATCGGCGGACTCGC--ATGGAGGAAGTAACCG-AGCAGGGCGTCCGCGTCACCGCGGGCCGTCCTCCGAGCGAG---CCCTCATCCGTTACAGACGCATTTGACTCTCGTCCGATGGACGCTGCAGCCGGTCTGGAAGTTGCCAGCGCGACGGCCTGAAGGCCGGGARAC-GGCAGGTGGCCGGTGGCCCAGCTTCG-TGCGGGTCGTCGGTTGT--TARCCATCCCGGTAGTGGCTCGTCTCCAGACCGAGGTGT-CGCGAGTCCCTTCG--GGGACTCCTCGTCTCCACCCC----AGAGTY-GCGGGACAGGGCACGGCCAGGGTTCTT-CACCGTCAT----CCTCCGCACTGGCACGGAGTGGGCGGAAGACACGGAGACCCGA--ACCSCCGTGACGGA--TCCGCGGGGGATCGGAGGCTGGCAGCGAGTAGGTCGGTCACCCACCCGACCCGTCTTGAAACACGGACCAAGGAGTCTAACATGTGCGCGAGTCAGTGGGTCTTGAACAGGCCCACGGGCGCAATGAAAGCGAAGGGTCCGCC-RAGYGCG-G---CCCGAGCGGGGATCCCCTTACTGTGT-GTTTGGGGCGCACCCGCGACCCGTCCTATCCGGCTCGCCGGTTGGGCGGAGTTTGAGCGTACACGTTGGGACCCGAAAGATGGTGAACTATGCCCGGACAGGACRAAGCCAGAGGAAACTCTGATGGAGGTCCGCAGCGGTTCTGACGTGCAAATCGATCGTCAGATCCGGGTCTAGGGG----------------------------------------------------------------------------------------------------------------------------------------------------------------------------------------------------------------------------------------------------------------------------------------------------------------------------------------------------------------------------------------------------------------------------------------------------------------------------------------------------------------------------------------------------------------------------------------------------------------------------------------------------------------------------------------------------------------------------------------------------------------------------------------------------------------------------------------------------------------------------------------------------------------------------------------------------------------------------------------------------------------------------------------------------------------------------------------------------------------------------------------------------------------------------------------------------------------------------------------------------

>Canama hinnulea JXZ089+JXZ161

------------------------------------------------------------------------------------------------------------------------------------------------------------------ACACTTTTAAAAAAGAAAGACGATAAGACCCTGTCGAACTTAACTTCAGTTTAACTGGGGTAGTTAATTAATTATTATTTTAATATAAATTAATTCATAGAAATCCAAAT-CATCTGATAAATAAGTTACCACAGGGATAACAGCGCAATTATATTTTAAAGCTCTTATTTAAAAAATAGATTACGACCTCGATGTTGAATTAATAATCC--CTTTTC----AAGCAAATTTGAAATTCAGGAAGTCTGTTCGACTTTTAAAAAATTACATGATTTGAGTTCAGACCGGCATAAGCCAGGTCGGCTTCTATCTTCTAAATTAATTTTTACAGTACGAAAGGACCTATTAATATAATATTATTATAAT-ATTGGCAGATTAATGCATTAGAATTAGAATCTAAATAATCTATTATATTATTTATAACAAATTATATCTTAATTTCAATAAGAATTTTAGTAAGAGTTGCTTTTTATACTATCTTAGAACGTAAAATTTTAGGATATATTCAAATTCGTAAAGGGCCTAATAAAGTAGGATTAATAGGGATTCTCCAACCTTTTAGAGATGCTTTAAAACTTTTCAATAAAAATCTTCTTCCTTCGGAACATATAAATATTGTTATATCATATATAACTCCAGCATTATCTTTAATGATTAGATTACTACTAATTCCTATTATTTTATTT------------------------------------------------------------------------------------------------------------GTACGGCGAGCGAAAAGGGATGAGCCCAGCGCCGAATCCCCGGCC---TCCGGTCGTGGGAAATGTGGCGTTTAGGAGTGAGCGTGTCGGGTTCGGAGGCGGTGGCAAGTCCCCCTGACCGGGGCCTGTCCGTAGCGGGTGCGAGACCCATAGCCGCCGCTCCCGT-CCCGTATGCGATCTCCTTGGAGTCGGTTTGCTTGGGAGTGCAGACCTAATCGGGTGGTAAACTCCACCTAAGGCTAAATACCGCCGTGAGACCGATAGCAAACAAGTACCGTGAGGGAAAGTTGCAAAGAACTTTGAAGAGAGAGTTCAAGAGTACGTGAAACTGCTCAGAGGTAAACGGGTGGGCCCTCGAAATCCTGTGGCGAGAGGATTCAGTCTGGATCGGCGGACTCGC--ATGGGGGAAGTAACCG-GGAAGGGCGTCCGCGTCACCGCGGGCCGTCCTCCGAGCGAG---CCCTCATCCGTTACAGACGCATTTGACTCTCGTCCGATGGACGCTGCAGCCGGTCTGGAAGTTGCCAGCGCGACGGCCTGAAGGCCGGGAGCC-GGCAGGTGGCCGGTGGCTCCGCTTCG-TGCGGGTCGTCGGTTGT--TAGCCATCCCGGTAGTGGCTCGTCTCCAGACCGAGGTGT-CGCGAGTCCCTTCG--GGGACTCCTCGTCTCCACCCC----AGAGTC-GCGGGACAGGGCACGGCCAGGGTTCTT-CACCGTCAT----CCTCCGCACTGGCACGGAGTGGGCGGAAGACACGGAGACCCGA--ACCGCCGTGACGGA--TCCGCGGGGGATCGGAGGCTGGCAGCGAGTAGGTCGGTCACCCACCCGACCCGTCTTGAAACACGGACCAAGGAGTCTAACATGTGCGCGAGTCARTGGGTCTTGAACAGGCCCACGGGCGCAATGAAAGCGAAGGGTCCGCC-GAGCGCG-G---CCCGAGCGGGGATCCCCTTACTGTGT-GTYTGGGGCGCACCCGCGACCCGTCCTATCCGGCTCGCCGGTTGGGCGGAGTTTGAGCGTACACGTTGGGACCCGAAAGATGGTGAACTATGCCCGGACAGGA---------------------------------------------------------------------------------------------------------------------------------------------------------------------------------------------------------------------------------------------------------------------------------------------------------------------------------------------------------------------------------------------------------------------------------------------------------------------------------------------------------------------------------------------------------------------------------------------------------------------------------------------------------------------------------------------------------------------------------------------------------------------------------------------------------------------------------------------------------------------------------------------------------------------------------------------------------------------------------------------------------------------------------------------------------------------------------------------------------------------------------------------------------------------------------------------------------------------------------------------------------------------------------------------------------------------------------

>Canama triramosa JXZ277

----------------------------AGA-TTAAATAGCCGCAATTGCGTGCTAAGGTAGCATAATCATATTGTCTTTTAAATAAGGACTAGAACAAAAGATTTTACATTTCAACTTTTTTATTTGAATTAAAATTTAAATTTATTTTAAATGTAAAAAGACATTTCTAAAAAAGAAAGACGACAAGACCCTATCGAACTTTACTTCAGTTTAACTGGGGCAGTTAATTAATTATTATTTTAATATAGATAATTATATACAAATCCATAA--AACTGATAAATCAGTTACCGTAGGGATAACAGCGCAATTAAATTTTAAAGCTCTTATTTAAAAAATAGATTACGACCTCGATGTTGAATTAATTACC---TATTTT----AAGCAAATTTTAAAA-AAGAAAGTCTGTTCGACTTTTAATAAATTACATGATTTGAGTTCAGACCGGKGTAAGCCAGGTCGGTTTCAATCTTCTAAAAAGTTTTTAGTAGTACGAAAGGACCTTAAGATATAATTAAATTAATTA-ATTGGCAGAGAAATGCATTAGAATTAGAATCTAAATAAACTTTTATATTATTTATTACTAATTATATTACAGTAAGAATTAGAATCCTTATTAGAGTAGCCTTTTATACTATTTTAGAACGTAAGATTCTAGGATACATTCAAATTCGTAAAGGTCCTAATAAAGTAGGGTTAATAGGAATCCTTCAACCTTTTAGAGATGCTCTAAAACTTTTTAATAAAAATTTATACATTTCAGAAAATATAAATTTCATTTTATCTTTATTAACTCCAGCAATATCTCTTCTTATTAGAATTTTACTAATTCCTATTATTTCTTATGATAAAATATCACTATATGATAATAAGCACAATATTTTATTATTTTTTATTTTATCAAGAATAGCAGTTTATTCAATATTATTAATTGGATGATCTGCTAA-------------------------------------------------------------------------------------TGAGCGTGTCGGGTTCGGATGCGGCGGCAAGTCCCCCTGACCGGGGCCTGTCCGTAGCGGGTGCGAGGCCCATAGCCGCCGCGCCCGT-CCCGAATGCGATCTCCTGGGAGTCGGGTTGCTTGGGAGTGCAGCCCTAATCGGGTGGTAAACTCCACCTAAGGCTAAATACCGCCGTGAGACCGATAGCAAACAAGTACCGTGAGGGAAAGTTGCAAAGAACTTTGAAGAGAGAGTTCAAGAGTACGTGAAACTGCTCAGAGGTAAACGGGTGGGCCCTCGAAATCCTGTGGCGAGAGGATTCAGTCTGGATCGGAGGATTCCGTTTGGGCGGAAGTAACCG-CGCAGGGCGTCCGCACCACTGCGGACCGTCCGCCTTG--GA---ACCTCTCCCGTGACAGACGCATTTGACTCTCGTCCGATGGACGCTGCAGCCGGTTTGGAAGTTGCCAGCGCGACGGCCTGAAGGCCGGGAGATAGGCAGGTGGCCGGTGGCTC-GTCTCG-TGCGGGTCGTCGGTTGT--TAGCCTTCCCGGCAGTGGCTCGTCTCCAGACCGAGGTGT-CGCGAGTCCCTTAAC-GGGACTCCTCGTCTCCACCCC----AGAGTC-GTGGGACAGGGCACGGACCGGG-TCTT-CGCCGTCGA----CCTCCGCACTGGCACGGAGTGGGCGGAAGATTCGGAGACCCAA--TCCGCCGTGACGGA--CCCGCGGGGGATCGGAGGCTCGCAGCGAGTAGGTCGGTCACCCACCCGACCCGTCTTGAAACACGGACCAAGGAGTCTAACATGTGCGCGAGTCAGTGGGTCTTGAATAGGCCCACAGGCGCAATGAAAGTGAAGGG-CTTCC-GAGCGTT-G---CCCGAGTGGGGATCCCTGCACCCCG--GTGCGGGGCGCACCCACGACCCGTCCATGCCGGCTCGCCGGTGGGGCGGAGTCTGAGCGTACACGTTGGGACCCGAAAGATGGTGAACTATGCCCGGACAGGACGAGGCCAGAGGAAACTCTGGTGGAGGTCCGCAGCGGTTCTGACGTGCAAATCGATCGTCAGATCCGGGTCTAGGGGC---------------------------------------------------------------------------------------------------------------------------------------------------------------------------------------------------------------------------------------------------------------------------------------------------------------------------------------------------------------------------------------------------------------------------------------------------------------------------------------------------------------------------------------------------------------------------------------------------------------------------------------------------------------------------------------------------------------------------------------------------------------------------------------------------------------------------------------------------------------------------------------------------------------------------------------------------------------------------------------------------------------------------------------------------------------------------------------------------------------------------------------------------------------------------------------------------------------------------------------------------

>Canama limagigi 11013 SAJ309

-------------------------------------------------------------------------------------------------------------------------------------------------------------------------------------------------------------------------------------------------------------------------------------------------------------------------------------------------------------------------ATTAATTATC---TAGTTA----AAGCAAATTTTAAAA-AAGAAAGTCTGTTCGACTTTTAATAAATTACATGATTTGAGTTCAGACCGGTGTAAGCCAGGTCGGTTTCAATCTTCTAGTTTCTTTTTAACAGTACGAAAGGACCTTAAAATATAATTTAATTAATTTAATTGGCAGATAAATGCATTAGAATTAGAATCTAAAAAAACTCCAATTCTATTTATTTCCAACTATATTATTATTTCTATCAGAATTCTAATCAGCGTAGCCTTCTATACTATTTTAGAGCGTAAAATCTTAGGGTATATTCAAATCCGGAAAGGCCCTAATAAAGTAGGAATATTAGGGATTCTTCAACCATTTAGAGACGCACTAAAACTTTTTAATAAAAATCTATATCTTTCAGAAACCATAAATTTTATCTTATCACTATCAACTCCAGCAATTTCCCTTTTAATTAGAATCTTATTAATCCCTATCATTTCTTACAATTATATATCATTATACGATAATAAACATAATATCCTCTTATTTTTTATTCTATCAAGAATAGCTGTTTATTCAATTTTATCAATTGGATGATCAGCTAA--------------------------------------------------------------------------------------------------------------------------------------------------------------------------------------------------------------------------------------------------------------------------------------------------------------------------------------------------------------------------------------------------CAGTCTGGATCGGAGGATTCCG--TGGGCGGAAGTAACCG-GGCAGGGCGTCCGCATCACTGCGGGCCGTCCGCCCTG--GA---ACCTCAGCCGTGACAGACGCATTTGACTCTCGTCCGATGGACGCTGCAGCCGGTTTGGAAGTTGCCAGCGCGACGGCCTGAAGGCCGGGAGACAGGCAGGTGGCCGGTGGCTC-GCCTCG-TGCGGGTCGTCGGTTGT--TAGCCTTCCCGGTAGTGGCTCGTCTCCAGACCGAGGTGT-CGCGAGTCCCTTCCT-GGGACTCCTCGTCTCCTCCCC----AGAGTC-GTGGGACAGGGCGCGGCCTGGG-TCTT-CGCCGTCGA----CCTCCGCACTGGCACGGAGTGGGCGGAAGAGACGGAGACCCTA--ACCGCCGCGACGGA--TCCGCGGGGGACTGGAGGCTCGCAGCGAGTAGGTCGGTCACCCACCCGACCCGTCTTGAAACACGGACCAAGGAGTCTAACATGTGCGCGAGTCAGTGGGTCTTGAACAGGCCCACGGGCGCAATGAAAGCGAAGGG-CTTCT-CCACGTT-G---CCCGAGTGGGGATCCCCGCACCTCGC-GTGTGGGGCGCACCCACGACCCGTCCATTCCGGCTCGCCGGTCGGGCGGAGTTTGAGCGTACACGTTGGGACCCGAAAGATGGTGAACTATGCCCGGACAGGACGAGGCCAGAGGAAACTCTGGTGGAGGTCCGCAGCGGTTCTGACGTGCAAATCGATCGTCAGATCCGGGTCTAGG---------TTGATCTGCTATGGTGGGGACGGCTATAAGAGTATTGATTCGAATGGAATTAGGTCAAACTGGTAATTTTTTAGGAAATGATCATTTGTATAATGTTATTGTTACTGCTCATGCTTTTGTTATAATTTTTTTTATGGTTATACCTATTATGATTGGTGGTTTTGGGAACTGATTAGTTCCTTTAATATTAGGGGCTCCGGATATAG--CTTTTCCTCGAATAAATAATCTTAGTTTTTGGCTATTACCTCCTTCTTTGATATTATTATTTATTTCTTCTATAGCGGAAATAGGAGTAGGTTCAGGGTGAACTGTTTATCCCCCACTAGCTTCTATTGTGGGACACAATGGAAGATCAGTAGATTTTGCTATTTTTTCTTTACATTTGGCTGGGGCTTCTTCTATTATAGGGGCTATTAATTTTATTTCTACGGTTATTAATATGCGATCTATTAGAATATCTATGGATAAAATTACTTTATTTGTGTGATCGGTGGTAATTACGGCTGTGTTGTTATTATTATCTTTACCTGTGTTAGCGGGTGCTATTACTATATTGTTAACAGATCGAAATTTTAATACTTCTTTTTTTGATCCTGCGGGAGGGGGAGATCCTATTTTATTTCAACATTTATTT---------------------------------------------------------------------------------------------------------------------------------------------------------------------------------------------------------------------------------------------------------------------------------------------------------------------------------------------------------------------------------------------------------------------------------------------------------------------------------------------------------------------------------------------------------------------------------

>Canama horobi 11034 SAJ455

-------------------------------------------------------------------------------------------------------------------------------------------------------------------------------------------------------------------------------------------------------------------------------------------------------------------------------------------------------------------------------------------------------------------------------------------------------------------------------------------------------------------------------------------------------------------------------------------------------------------------------------------------------------------------------------------------------------------------------------------------------------------------------------------------------------------------------------------------------------------------------------------------------------------------------------------------------------------------------------------------------------------------------------------------------------------------------------------------------------------------------------------------------------------------------------------------------------------------------------------------------------------------------------------------------------------------------------------------------------------------------------GATTCAGTCTGGATCGGAGGATTCCG--CGGGCGGAAGCAACCG-GGCAGGGCGTCCGCATCACTGCGGGCCGTCCGCCTCG--GA---ACCTCAGCCGTGACAGACGCATTTGACTCTCGTCCGATGGACGCTGCAGCCGGTTTGGAAGTTGCCAGCGCGACGGCCTGAAGGCCGGGAGACAGGCAGGTGGCCGGTGGCTC-GCCTCG-TGCGGGTCGTCGGTTGT--TAGCCTTCCCGGTAGTGGCTCGTCTCCAGACCGAGGTGT-CGCGAGTCCCTTCCT-GGGACTCCTCGTCTCCTCCCC----AGAGTC-GTGGGACAGGGCACGGCCAGGG-TCTT-CGCCGTCGA----CCTCCGCACTGGCACGGAGTGGGCGGAAGAGACGGAGACCCTA--ACCGCCGTGACGGA--CCCGCGGGGGACCGGAGGCTCGCAGCGAGTAGGTCGGTCACCCACCCGACCCGTCTTGAAACACGGACCAAGGAGTCTAACATGTGCGCGAGTCAGTGGGTCTTGAACAGGCCCACGGGCGCAATGAAAGCGAAGGG-CTTCT-CCACGTT-G---CCCGAGTGGGGATCCCCGCGCCTCGC-GCGCGGGGCGCACCCACGACCCGTCCATGCCGGCTCGCCGGTCGGGCGGAGTTTGAGCGTACACGTTGGGACCCGAAAGATGGTGAACTATGCCCGGACAGGACGAGGCCAGAGGAAACTCTGGTGGAGGTCCGCAGCGGTTCTGACGTGCAAATCGATCGTCAGATCCGGGTCTAGG---------ATGATCTGCTATAGTAGGTACTGGTATAAGTGTTTTGATTCGAATGGAGTTAGGTCAAACTGGTAATTTTTTAGGAAATGATCATTTGTATAATGTTATTGTTACTGCTCATGCTTTTGTTATGATTTTTTTTATGGTAATACCTATTATAATTGGTGGTTTTGGAAATTGATTGGTTCCGTTGATGTTAGGGGCTCCTGATATAG--CTTTTCCTCGAATAAATAATTTGAGTTTTTGGTTATTACCCCCTTCTTTAATATTATTGTTTATTTCTTCAATGGTGGAAATAGGAGTTGGTTCTGGGTGAACTGTTTATCCTCCTTTAGCTTCAATTATGGGACATAATGGAAGATCGGTGGATTTTGCTATTTTTTCTTTACATTTAGCTGGAGCTTCTTCGATTATGGGGGCTATTAATTTTATTTCTACAGTTATTAATATACGATCTGTAAATATGTCTATAGATAAAATTACTTTATTTGTGTGGTCAGTAGTTATTACAGCTGTTTTATTATTATTATCTTTACCTGTATTAGCAGGTGCTATTACTATATTATTAACGGATCGAAATTTTAATACCTCTTTTTTTGACCCTGCAGGGGGAGGAGACCCTATTTTATTTCAACATTTATTTTGATTTTTTGGTCACCCTG--------------------------------------------------------------------------------------------------------------------------------------------------------------------------------------------------------------------------------------------------------------------------------------------------------------------------------------------------------------------------------------------------------------------------------------------------------------------------------------------------------------------------------------------------------------

>Bathippus madang 10807 SAJ308

-------------------------------------------------------------------------------------------------------------------------------------------------------------------------------------------------------------------------------------------------------------------------------------------------------------------------------------------------------------------------ATTAATAACC---TTTTTA----AAGCAATTTTTAGAA-AAGGAAGTCTGTTCGACTTTTAACAAATTACATGATTTGAGTTCAGACCGGTATAAGCCAGGTCGGCTTCAATCTTCTAAAAACTCTTTTACAGTACGAAAGGACCTAAAAATATAATTTAATTAATTA-ATTGGCAGATTAATGCATTAGAATTAGAATCTAATAAAACTTCTATTCCATTCTTTCTTAATTATATTCTAATTCTGATTAGAATCCTAATCAGTGTAGCATTCTACACTATTCTAGAACGAAAAATTCTAAGATATATTCAAACCCGAAAAGGACCTAATAAAGTAGGACTTATAGGGATTCTTCAACCTTTCAGAGATGCCTTAAAACTTTTTAATAAAAATTTATCAATATCCGAAAATATAAATTTAATTTTATCTTCCTTAACTCCAGCTATATCTTTAATAATTAGAATATTATTAATTCCTATTATCCTCTATGATTCCATATCCCTATATGATAACAAACATAACATTTTACTATTTTTCATTTTATCTAGAATTGCAGTATATTCAATATTATTAATTGGGTGATCCGCAAA----------------------------------------------------------------------------------------------------------------------------------------------------------------------------------------------------------------------------------------------------------------------------------------------------------------------------------------------------------------------------------------------------------------------------------------------------------------------------------------------------------------------------------------------------------------------------------------------------------------------------------------------------------------------------------------------------------------------------------------------------------------------------------------------------------------------------------------------------------------------------------------------------------------------------------------------------------------------------------------------------------------------------------------------------------------------------------------------------------------------------------------------------------------------------------------------------------------------------------------------------------------------------------------------------------------------------------------------------------------------------------------------------------------------------------------------------------------------------------------------------------------------------------------------------------------------------------------------------------------------------------------------------------------------------------------------------------------------------------------------------------------------------------------------------------------------------------------------------------------------------------------------------------------------------------------------------------------------------------------------------------------------------------------------------------------------------------------------------------------------------------------------------------------------------------------------------------------------------------------------------------------------------------------------------------------------------------------------------------------------------------------------------------------------------------------------------------------

>Bathippus madang 10808 SAJ304

-------------------------------------------------------------------------------------------------------------------------------------------------------------------------------------------------------------------------------------------------------------------------------------------------------------------------------------------------------------------------ATTAATAACC---TTTTTA----AAGCAATTTTTAGAA-AAGGAAGTCTGTTCGACTTTTAACAAATTACATGATTTGAGTTCAGACCGGTATAAGCCAGGTCGGCTTCAATCTTCTAAAAACTCTTTTACAGTACGAAAGGACCTAAAAATATAATTTAATTAATTA-ATTGGCAGATTAATGCATTAGAATTAGAATCTAATAAAACTTCTATTCCATTCTTTCTTAATTATATTCTAATTCTGATTAGAATCCTAATCAGTGTAGCATTCTACACTATTCTAGAACGAAAAATTCTAAGATATATTCAAACCCGAAAAGGACCTAATAAAGTAGGACTTATAGGGATTCTTCAACCTTTCAGAGATGCTTTAAAACTTTTTAATAAAAATTTATCAATATCCGAAAATATAAATTTAATTTTATCTTCCTTAACTCCAGCTATATCTTTAATAATTAGAATATTATTAATTCCTATTATCCTCTATGATTCCATATCCCTATATGATAACAAACATAACATTTTACTATTTTTCATTTTATCTAGAATTGCAGTATATTCAATATTATTAATTGGGTGATCCGCAAA---------------------------------------------------------------------------------------------------------------------------------------------------------------------------------------------------------------------------------------------------------------------------------------------------------------------------------------------------------------------------------------------GGATTCAGTCTTGAGCGGAGGACTTC---CCGGAGGAAAAAACCATAGCAGGGCGTCCGCGTCG-CGCGGGCCGTCCTCTGGGT-CG---ACCTCTGCCGCCAAAGACGCATTTGACTCTCGCCCCGAGGACGCTGCAGCCGGTCTGGAAGTTGCCAGCGCGACGGCCTGAGGGCCGGGAACCGGGCAGGTGGCCGGTGGCTC-GCCGCG-TGCGGGTCGTCGGTTGT--TAGCCCTCCCGGCAGTGGCTCGTCTCCAGACCGAGGTGT-CGCGAGTCCCTTCG--GGGACTCCTCGTCTCCACCCC----AGAGTC-GCGGGACAGGGCACGGACCGGG-TCTT-CGCCGTCAA----CCTCCGCACTGGCACGGAGTGGGCGGAAGACACGGAGACCCAA--TCCGCCGTGACGGA--CCCGCGGGGGACCGGAGGCTGGCAGCGAGTAGGTCGGTCACCCACCCGGCCCGTCTTGAAACACGGACCAAGGAGTCTAACATGTGCGCGAGTCAGTGGGTCTTGAACAGGCCCACGGGCGCAATGAAAGCGAAAGG-CCGCC-GAGCGCG-G---CCCGAGTGGGGATCCTCGCCCTTCGCGGGGCGGGGCGCACCCACGACCCGTCCTATCCGGCTCGCCGGTTGGGCGGAGTCTGAGCGTACACGTTGGGACCCGAAAGATGGTGAACTATGCCCGGACAGGACGAGGCCAGAGGAAACTCTGGTGGAGGTCCGCAGCGGTTCTGACGTGCAAATCGATCGTCAGATCCGGGTCTAGGGG--------TGATCTGCTATAGTGGGTACAGCTATAAGAGTTTTAATTCGAATAGAATTAGGACAAACTGGAAATTTTTTAGGAAATGATCATTTGTATAATGTTATTGTTACTGCTCATGCTTTTGTTATGATTTTTTTTATAGTTATACCTATTATAATTGGAGGTTTTGGAAATTGATTGGTCCCTTTAATGTTAGGTGCCCCGGATATAG--CTTTTCCTCGTATGAATAATTTAAGCTTTTGATTGTTACCCCCATCTTTAATATTGTTGTTTGTTTCTTCTATATCTGAAATAGGGGTAGGTTCTGGATGAACGGTTTATCCCCCCTTAGCTTCCATTATTGGTCATAATGGAAGCTCAGTAGATTTTGCTATTTTTTCTTTACATTTGGCTGGGGCATCTTCTATTATAGGGGCTATTAATTTTATTTCTACTGTTATTAACATACGATCTGTTGATATATCGATGGATAAAATTACTTTGTTTGTATGGTCTGTAATTATTACAGCTGTTTTATTGTTGTTATCTTTACCAGTTTTAGCTGGAGCTATTACTATATTACTTACAGATCGAAATTTCAATACTTCTTTTTTTGATCCTGCAGGAGGGGGGGATCCGGTGTTATTTCAACATTTATTT---------------------------------------------------------------------------------------------------------------------------------------------------------------------------------------------------------------------------------------------------------------------------------------------------------------------------------------------------------------------------------------------------------------------------------------------------------------------------------------------------------------------------------------------------------------------------------

>Canama wanita SAJ148

---------------------------------------------------------------------------------------------------------------------------------------------------------------------------------------------------------------------------------------------------------------------------------------------------------------------------------------------------------------------------------------------------------------------------------------------------------------------------------------------------------------------------------------------------------------------------------------------------------------------------------------------------------------------------------------------------------------------------------------------------------------------------------------------------------------------------------------------------------------------------------------------------------------------------------------------------------------------------------------------------------------------------------------------------------------------------------------------------------------------------------------------------------------------------------------------------------------------------------------------------------------------------------------------------------------------------------------------------------------------------------------------------------------------------------------------------------------------------------------------------------------------------------------------------------------------------------------------------------------------------------------------------------------------------------------------------------------------------------------------------------------------------------------------------------------------------------------------------------------------------------------------------------------------------------------------------------------------------------------------------------------------------------------------------------------------------------------------------------------------------------------------------------------------------------------------------------CTGGGTCTGCTATAGTAGGTACAGCTATAAGAGTATTGATTCGAATGGAATTAGGTCAAACTGGTAATTTTTTAGGAAATGATCATCTATATAATGTTATTGTTACTGCTCATGCTTTTGTTATAATTTTTTTTATAGTAATGCCTATTATAATTGGTGGGTTTGGTAATTGATTAGTTCCTTTAATATTAGGGGCTCCAGATATAG--CTTTTCCTCGTATAAATAATCTTAGTTTTTGATTATTACCTCCTTCTTTAATATTATTATTTATTTCTTCTATGGTGGAGATAGGGGTAGGTTCAGGGTGAACTGTATATCCTCCTTTAGCTTCTATTGTAGGACACAATGGAAGATCAGTAGATTTTGCTATTTTTTCTTTACATTTAGCTGGTGCTTCTTCTATTATGGGGGCTATTAATTTTATTTCTACGGTTATTAATATACGATCTGTTAGAATAAGTATAGATAAGATTACTTTATTTGTATGATCAGTAGTAATTACAGCTGTGTTATTATTATTGTCTTTACCAGTATTGGCAGGTGCTATTACTATATTATTAACAGATCGAAATTTTAATACTTCTTTTTTTGATCCTGCAGGTGGAGGGGATCCTATTTTATTTCAACATTTATTT---------------------------------------------------------------------------------------------------------------------------------------------------------------------------------------------------------------------------------------------------------------------------------------------------------------------------------------------------------------------------------------------------------------------------------------------------------------------------------------------------------------------------------------------------------------------------------

# MODEL SELECTION

SMS

Input alignment	: align_rename.phy

Data type	: DNA

Number of taxa	: 20

Number of sites	: 3319

Number of branches : 37

Criterion	: AIC

~~~~~~~~~~~~~~~~~~~~~~~~~~~~~~~~~~~~~~~~~~~~~~~~~~~~~~~~~~~~

Step 1 : Set a fixed topology	AIC=32192.53084

~~~~~~~~~~~~~~~~~~~~~~~~~~~~~~~~~~~~~~~~~~~~~~~~~~~~~~~~~~~~

Step 2 : Select the best decoration

AIC=32192.53084	decoration : '+G+I'

~~~~~~~~~~~~~~~~~~~~~~~~~~~~~~~~~~~~~~~~~~~~~~~~~~~~~~~~~~~~

Step 3 : Select the best matrix	AIC=0	matrix : 'GTR'

~~~~~~~~~~~~~~~~~~~~~~~~~~~~~~~~~~~~~~~~~~~~~~~~~~~~~~~~~~~~

Step 4 : Select the best final decoration

AIC=32192.53084	decoration : '+G+I'

~~~~~~~~~~~~~~~~~~~~~~~~~~~~~~~~~~~~~~~~~~~~~~~~~~~~~~~~~~~~

Selected model	: GTR +G+I

~~~~~~~~~~~~~~~~~~~~~~~~~~~~~~~~~~~~~~~~~~~~~~~~~~~~~~~~~~~~

Substitution model	: GTR

Equilibrium frequencies	: ML optimized

Proportion of invariable sites	: estimated (0.366)

Number of substitution rate categories	: 4

Gamma shape parameter	: estimated (0.470)

~~~~~~~~~~~~~~~~~~~~~~~~~~~~~~~~~~~~~~~~~~~~~~~~~~~~~~~~~~~~

Additional step : Infer tree with model : 'GTR +G+I+F'
